# An improved V-Net lung nodule segmentation model based on pixel threshold separation and attention mechanism

**DOI:** 10.1038/s41598-024-55178-3

**Published:** 2024-02-27

**Authors:** Xiaopu Ma, Handing Song, Xiao Jia, Zhan Wang

**Affiliations:** 1 School of Computer Science and Technology, Nanyang Normal University, Nanyang, 473061 China; 2 School of Life Sciences and Agricultural Engineering, Nanyang Normal University, Nanyang, 473061 China

**Keywords:** Image processing, Computer science

## Abstract

Accurate labeling of lung nodules in computed tomography (CT) images is crucial in early lung cancer diagnosis and before nodule resection surgery. However, the irregular shape of lung nodules in CT images and the complex lung environment make it much more challenging to segment lung nodules accurately. On this basis, we propose an improved V-Net segmentation method based on pixel threshold separation and attention mechanism for lung nodules. This method first offers a data augment strategy to solve the problem of insufficient samples in 3D medical datasets. In addition, we integrate the feature extraction module based on pixel threshold separation into the model to enhance the feature extraction ability under different thresholds on the one hand. On the other hand, the model introduces channel and spatial attention modules to make the model pay more attention to important semantic information and improve its generalization ability and accuracy. Experiments show that the Dice similarity coefficients of the improved model on the public datasets LUNA16 and LNDb are 94.9% and 81.1% respectively, and the sensitivities reach 92.7% and 76.9% respectively. which is superior to most existing UNet architecture models and comparable to the manual level segmentation results by medical technologists.

## Introduction

**Figure 1:**
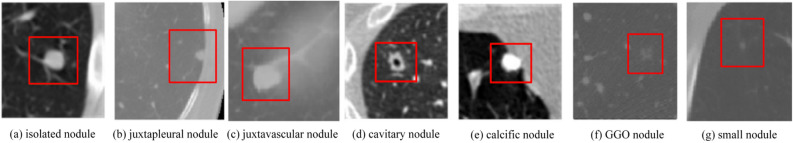
Legend of each morphological nodule.

Lung cancer is a common malignant tumor. According to the latest Global Cancer Research Association data, lung cancer has become one of the most deadly cancers worldwide^1^. Up to 2016, the global incidence and mortality rate of lung cancer has surpassed all other types of cancers, making it one of the most severe public health problems. This data shows lung cancer significantly threatens people’s health and social development^2^. The early imaging manifestation of lung cancer is the presence of lung nodule formation^3^. According to the different characteristics of lung nodules, they can be divided into seven categories: isolated nodule, juxta pleural nodule, juxta vascular nodule, cavitary nodule, calcific nodule, GGO nodule, and small nodule^4^. The clinical features of various lung nodules are shown in Fig. 1.

Due to the concealment of lung cancer, most patients have missed the best stage of treatment when they are diagnosed with lung cancer. Therefore, timely detection of lung cancer and effective treatment under scientific intervention are the best strategies to improve the survival rate of lung cancer patients. Recent research has shown that low-dose computed tomography (CT) scans can be used to determine the morphology of lung nodules, allowing for accurate screening of early-stage lung cancer to reduce the risk of death in lung cancer patients significantly^1^. Therefore, precise identification and segmentation of lung nodules have become a crucial part of the later treatment of lung cancer, and it is also a difficult point in clinical research. However, accurate segmentation and screening of many lung nodules is a massive test for clinicians ’ experience, technique, and energy. This leads to the lack of empirical misdiagnosis and missed diagnosis in manual lung nodular segmentation^5^. Although deep learning has made some achievements in lung nodules segmentation, there are still many problems:

- CT medical datasets are very limited, and an effective method for data augmentation of 3D medical image datasets is still lacking.
- The volume of lung nodules is small, its proportion in CT images is low, and the general 3D neural network model is challenging to extract the fine and hierarchical features of the lesion area. Hence the segmentation effect of the traditional operation is not good.

Based on the above disadvantages, the main contributions of this research are as follows:

1. In the data processing stage, a method suitable for 3D medical data augmentation is proposed to augment the dataset by doing slice reverse order on the cross-section and matching plane graph transformation, which has solved the problem of insufficient medical data samples.
2. In the model construction stage, we propose a separation module based on pixel thresholds and also integrate the channel and spatial attention mechanisms into the model. The model strengthens the ability to extract hierarchical features and the learning ability of important feature information, respectively, and improves the segmentation ability and accuracy of the improved model.

The structure of this paper is as follows: In the Related Works section, we summarize the research work and progress of 2D segmentation, multi-view 2D segmentation (pseudo-3D segmentation), and 3D segmentation according to different segmentation methods, and compare the advantages and disadvantages of various technologies. In the section of “Materials and methods”, we introduce the methods and advantages used in this paper in detail, which mainly include improved model construction, input layer design based on pixel threshold feature separation, downsampling layer integrated with 3D-CBAM attention, and activation function replacement. The Experiments and Results section mainly introduces the experimental dataset and its processing and augmentation, experimental hardware and software environment configuration, and model evaluation index. In addition, this section also includes performance comparison experiments between the methods used in this paper and other methods on the same dataset, ablation experiments of model improvement and data augmentation methods, and specific analysis of each experiment. Finally, we briefly summarize the proposed methods and future work directions in the conclusion part.

## Related works

In recent years, many deep learning methods have been proposed and applied to lung nodule segmentation, which can help doctors diagnose and treat lung cancer patients more accurately by learning a large amount of medical image data and extracting features of nodules from them to achieve automatic nodule segmentation while improving clinical efficiency and accuracy.

According to the different dimensions of input medical data, the segmentation of lung nodules based on deep learning can be divided into 2D segmentation for lung nodule slices and 3D segmentation for 3D medical volumes. For 2D segmentation works, Long et al.^6^ proposed the use of Full Connection Network (FCN) for image semantic segmentation, a network model that can accept image inputs of different sizes while enabling a large improvement in segmentation speed, providing a new idea for the task of lung nodule segmentation. In 2015, Ronneberger et al.^7^ proposed a UNet network based on encoder-decoder structure and applied to medical image data segmentation tasks. The architecture consists of a contracting path to capture context and a symmetric expanding path that enables precise localization. In addition, it creatively uses a method called Skip Connection to combine the low-level semantic information extracted from each layer of the encoder with the abstract semantic information of the decoder , which greatly improves the accuracy of segmentation. This work has been proved to significantly improve the accuracy of detection and segmentation of multiple biomedical images including pulmonary nodule segmentation. In 2017, Wang et al.^8^ presented a multi-view convolutional neural networks (MV-CNN) for lung nodule segmentation. The MV-CNN specialized in capturing a diverse set of nodule-sensitive features from axial, coronal and sagittal views in CT images simultaneously. The proposed network architecture consists of three CNN branches, where each branch includes seven stacked layers and takes multi-scale nodule patches as input. The average dice similarity coefficient (DSC) is 77.67%, and the average surface distance (ASD) is 0.24, which is superior to the traditional image segmentation method. Wang et al.^9^ proposed an improved UNet segmentation network based on parallel attention and Long Short-Term Memory (LSTM)^10^. The network model uses a mixed loss function, which effectively alleviates the problem of category imbalance and achieves better segmentation results. In 2020, Varma et al.^11^ proposed an improved U-Net network combining bidirectional cross-scale connection, weighted feature module (Bi-FPN)^12^, and improved activation function. They achieved a Dice similarity coefficient (DSC) of 82.82%. Li et al.^13^ proposed an encoder-decoder structure model based on Transformer, which creatively uses the Cross-Transform model and the bidirectional vertebral module to extract features and effectively achieve a more accurate segmentation effect. Ma et al.^14^ proposed a 2D Multi-level Dynamic Fusion Network (MDFN) to solve the problem of key space and edge detail loss in the process of pulmonary nodule segmentation. The network enriches the receptive field and captures multi-scale context information by constructing a multi-scale spatial and channel feature selection module (MSCFS). Finally, the test on the LUNA16 dataset reaches the level of DSC coefficient of 89.19%, which proves the effectiveness of the scheme. In 2021, Dutande et al.^15^ proposed a network model SquExUNet, which introduced attention mechanism based on U-Net, for the segmentation of pulmonary nodules, and obtained a DSC coefficient of 0.80 on LIDC, LNDb challenge dataset and purely independent Indian Lung CT Image Database (ILCID) clinical dataset. Sensitivity is 90.01%. In 2023, Deepajothi et al.^16^ proposed an improved segmentation model based on U-Net to accurately identify pulmonary nodules and determine their malignancy. The method is verified on the public dataset LUNA16, and the DSC coefficient of the segmentation result reaches 89%, which achieves reliable results. Hou et al.^17^, proposed a model named MSR-UNet, which is based on U-Net network model and integrates self-attention, multi-scale features and residual structure, and achieves a good Dice coefficient of 91.87% and an IoU of 86.8% on LIDC dataset. Tang et al.^18^ proposed a Res2Net50-based backbone network, which includes three modules : High-Level Feature Decoder Module (HDM), Low-Level Feature Decoder Module (LDM) and Complementary Module (CM). Through the recognition and fusion of high-level and low-level semantic information, more accurate edge segmentation is achieved. Finally, the DSC coefficient is 83.5% on the LUNA16 dataset. The segmentation accuracy with a sensitivity of 86.5% is higher than most existing methods. Tang et al.^19^ proposed a scale-aware-based multiattention-guided reverse network (SM-RNet), which extracts multi-scale features with channel and scale awareness, and mines detailed features through the scale-guided spatial attention (SSA) block and a new RE block attention mechanism. Finally, the DSC coefficients of the model on the public datasets FUSCC and LUNA16 reach 89.29% and 86.496%, respectively, and the model has strong robustness. Joshua et al.^20^ used the logical distribution model of three parameters in the feature extraction process and used the U-Net network structure as the benchmark model. The scheme achieved excellent results in the segmentation of pulmonary nodules. The DSC coefficient, sensitivity and specificity on the LUNA16 public dataset reached 97.3%, 96.5% and 94.1%, respectively, which exceeded most of the existing methods.

Although the above segmentation method based on the 2D convolutional neural network has achieved good accuracy through various improvements, such methods can only learn the semantic features of plane images, lack feature extraction ability for high-dimensional data features, and are sensitive to feature changes caused by graphic deformation.

Based on the problems of 2D segmentation, some researchers use the multi-view segmentation fusion method to make up for the defects of the 2D segmentation model. Among them, Zhang et al.^21^ proposed a 2.5D-based segmentation method for medical image segmentation. This method enables the network to have a deeper and broader architecture while retaining some 3D semantic information. The results of training and testing on the dataset of Liver and Tumor Segmentation Challenge (LiTS) show that the method has an accuracy close to 3D segmentation effect when the slice layer spacing is small. However, this method is greatly affected by the distance between slices. Zheng et al.^22^ proposed a model for 2.5D segmentation, which offered an association module for slices from different perspectives and designed a new loss function (Square Root Dice loss) to deal with the trade-off between Sensitivity and Specificity. Wardhana et al.^23^ explored the multi-view segmentation model’s model structure, parameter tuning, and the relationship between the number of network layers and the design. Networks are trained and tested by utilizing the dataset from the liver and tumor segmentation challenge (LiTS). The network performance was further evaluated by comparing the network segmentation with manual segmentation from nine technical physicians and an experienced radiologist. In 2023, Chen et al.^24^ proposed an end-to-end network called Fast Multiple Clipping Guided Attention (FMGA), which uses the fusion features in two directions of 2D slices to aggregate contextual feature information, and finally achieved good results. Ni et al.^25^ proposed a coarse-to-fine 2-stage framework consisting of the following 2 convolutional neural networks: a 3D multiscale U-Net used for localization and a 2.5D multiscale separable U-Net (MSU-Net) used for segmentation refinement. The proposed method achieved a Dice similarity coefficient (DSC) of 83.04% and an overlapping error of 27.47% on the dataset. The 2.5D multi-view segmentation model considers the speed of 2D segmentation and the three-dimensional feature information of 3D objects. However, it is still affected by the perspective direction of data slices and has a poor segmentation effect, and it is not suitable for object segmentation with strong spatial variation.

To address the deficiencies encountered in 2.5D segmentation, some scholars have tried to use 3D neural network architecture in semantic segmentation. Chen et al.^26^ proposed a model called ViT-V-Net, which uses Multi-Head Attention in ViT^27^ networks to relate long-range spatial features in medical images. To accurately learn the location features of lesions and counteract the loss of detailed localization information due to continuous downsampling, the authors combined the ViT-based image segmentation method with ConvNets to improve the recovery of detailed localization information and achieved superior performance. However, the technique uses more parameters and computer arithmetic power. Wang et al.^28^ proposed a multimodal medical segmentation model Med-DANet for the problem that 2D segmentation methods ignore the heterogeneity of image slice data in the segmentation process of 3D medical images (e.g. CT and MRI).The model based on adaptive model selection to achieve effective accuracy and efficiency trade-off. For each slice of the input 3D MRI volume, the proposed method learns a slice-specific decision by the Decision Network to dynamically select a suitable model from the predefined Model Bank for the subsequent 2D segmentation task.The proposed method finally achieved better segmentation results. Hatamizadeh et al.^29^ proposed a new architectural network UNEt TRansformers (UNETR), that utilizes a transformer as the encoder to learn sequence representations of the input volume and effectively capture the global multi-scale information, while also following the successful ”U-shaped” network design for the encoder and decoder. The transformer encoder is directly connected to a decoder via skip connections at different resolutions to compute the final semantic segmentation output. The model has demonstrated excellent performance on several medical datasets. Yu et al.^30^ found a 3D lung nodule segmentation network 3D Res U-Net that combines ResNet^31^ and UNet. The model replaces the ordinary convolution in the encoder and decoder structure with the residual module, greatly improving the segmentation performance. The segmentation effect is higher than the 3D U-Net based on the residual mechanism. Tyagi et al.^32^ proposed a lung nodule segmentation model CSE-GAN based on a GAN generation network^33^, which obtained better segmentation results by learning data distribution. The Dice similarity coefficient of the model reached 80.74%, and the sensitivity reached 85.46%. Hou et al.^34^ proposed a combination of CRF (Conditional Random Field)^35^ and 3D-UNet^36^. The Dice score of this method got 93.25% on the LUNA16 dataset.

However, 3D segmentation also has a few shortcomings, such as significant sample demand and insufficient accuracy of edge information for irregularly segmented objects. On this basis, this paper proposes an improved V-Net^37^ segmentation model, based on pixel threshold separation and attention mechanism, named Dig-CS-VNet, to overcome the above disadvantages.

## Materials and methods

### Dig-CS-VNet structure design

**Figure 2:**
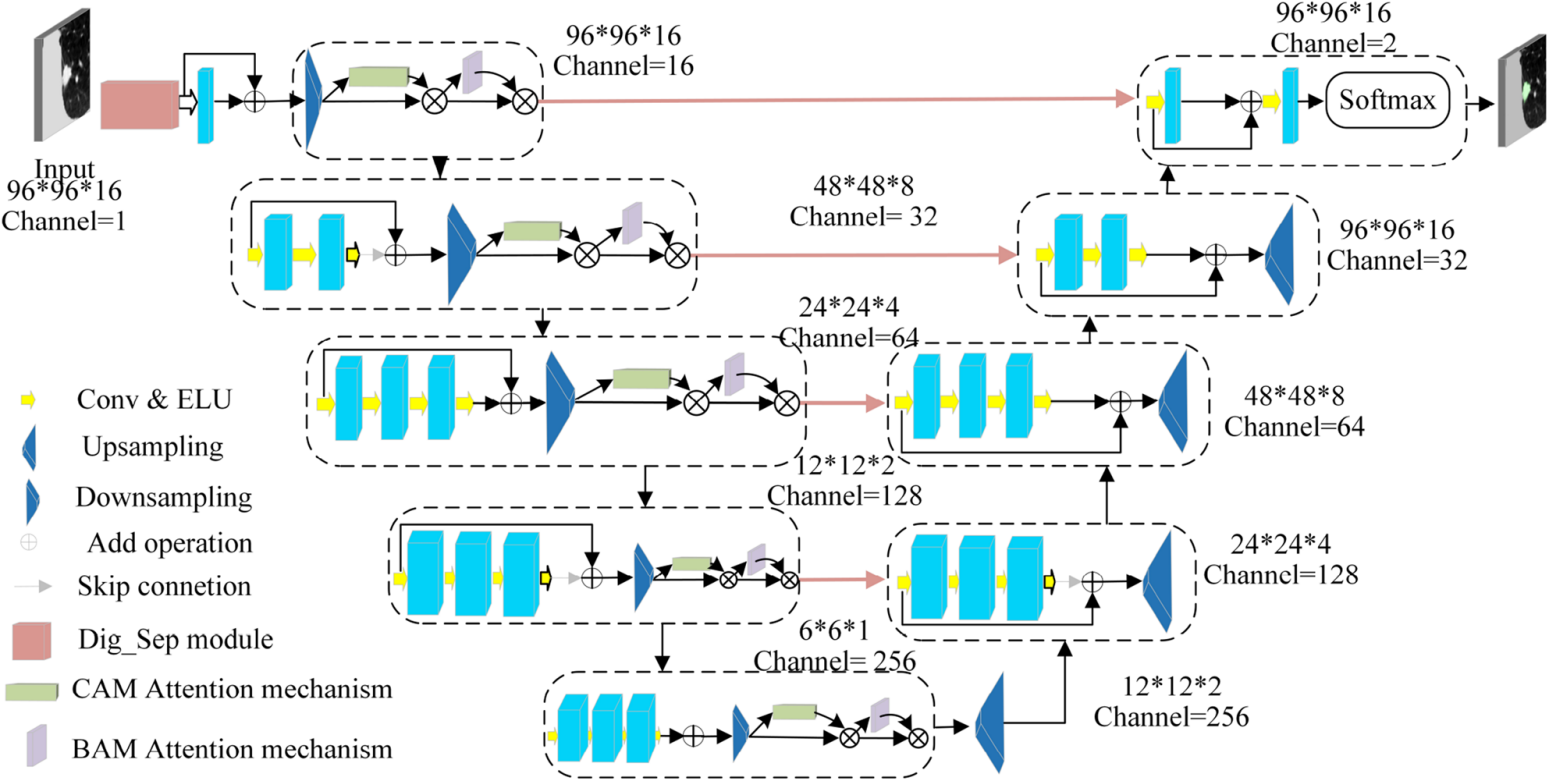
Dig-CS-VNet network architecture.

The improved V-Net model for lung nodule segmentation (Dig-CS-VNet) proposed in this paper is shown in Fig. 2. The cropped 3D CT image is first fed into the input layer, and the channel is expanded to 16 channels by the feature separation module (Dig_Sep) and the feature replication operation. To enable the encoder to be more fine-grained for specific semantic information acquisition and thoroughly learn the edge contour information of the nodule, we incorporate the Three-dimensional Convolutional Block Attention Module (3D-CBAM) attention module at the end of each downsampling layer. After four downsampling modules, the feature information is fed to the decoder. In this part, the feature image size of each output decoder is equal to the corresponding level encoder; the encoder splices the output features with the output features of the corresponding decoder as the input features of the next layer through a jump connection to fuse the underlying semantic information and achieve the purpose of fine segmentation.

### Dig_Sep module

Inspired by the adaptive pyramid module Adaptive Structural Pyramid Pooling(ASPP)^38^ to extract multi-scale features of images. In this paper, we propose a network module Digit_Sep for multi-level feature separation and extraction based on pixel threshold. Dig_Sep is a new type of portable network module that almost does not occupy additional computational overhead. This module can improve the extraction ability of specific features and effectively improve the segmentation accuracy of the network by learning the separate parts.

Usually, images are stored in the computer as pixel values. Each pixel corresponds to a gray value, indicating the degree of brightness of the point, usually expressed as an integer between 0 and 255. The segmentation task in medical images is usually to separate the regions of interest in the picture, and these regions have apparent differences in gray level. Therefore, by reasonably dividing the gray threshold of the original image matrix and then performing algebraic operations on images of different gray levels, the image semantic features under different thresholds can be separated to separate the regions of interest effectively. In addition, this method can also screen out some invalid interference factors and improve the accuracy of image segmentation.

**Figure 3:**
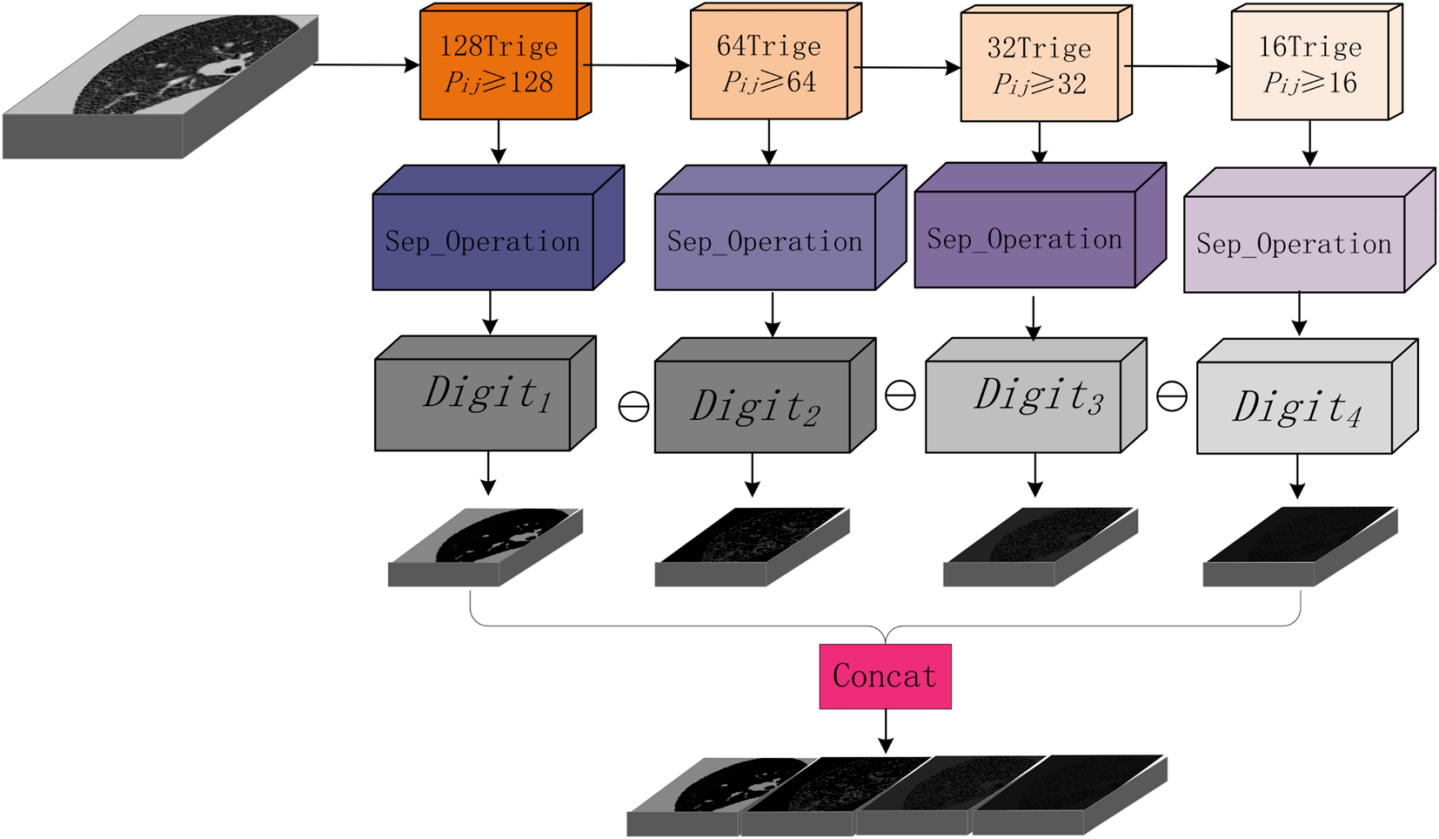
Dig_Sep block.

The specific operation of this module is as Fig. 3. Supposed the sample image is a gray image with a size of 96 $\times$ 96. The gray value of each pixel in the image matrix *P* corresponding to it is represented by the 8-bit binary number ${P_{ij}}$, and its number from high to low is ${p_1}$ , ${p_2}$ ,..., ${p_7}$ , ${p_8}$. We take the image matrix from high to low one by one and binarize it according to the threshold to obtain the digital feature map. For example, we take ${p_1}$ as the benchmark. If ${P_{ij}}\ge 27$, it is assigned to 128; otherwise, 0. Thus the first digital feature map ${Digit_1}$ is obtained. Then, we use the original image matrix to subtract the digital feature map, *P* $=$ *P* − ${Digit_1}$. Then, based on the ${p_2}$ bit, we repeat the above operation to obtain the second digital feature map ${Digit_2}$. Repeat the above steps to get eight digital feature maps finally. Finally, we select the retained digital feature map according to the specific characteristics of the gray data distribution as the input of subsequent image segmentation. Due to the different segmentation objects, the semantic information characteristics are different. Therefore, in the process, the representative feature layers with more representative feature information should be selected according to the specificity of the working object. In this work, because the high four bits of the separation feature map can best represent the texture features of the lung region, we only retain the high four bits of the separation layer as part of the input features of the subsequent model.

**Figure 4:**
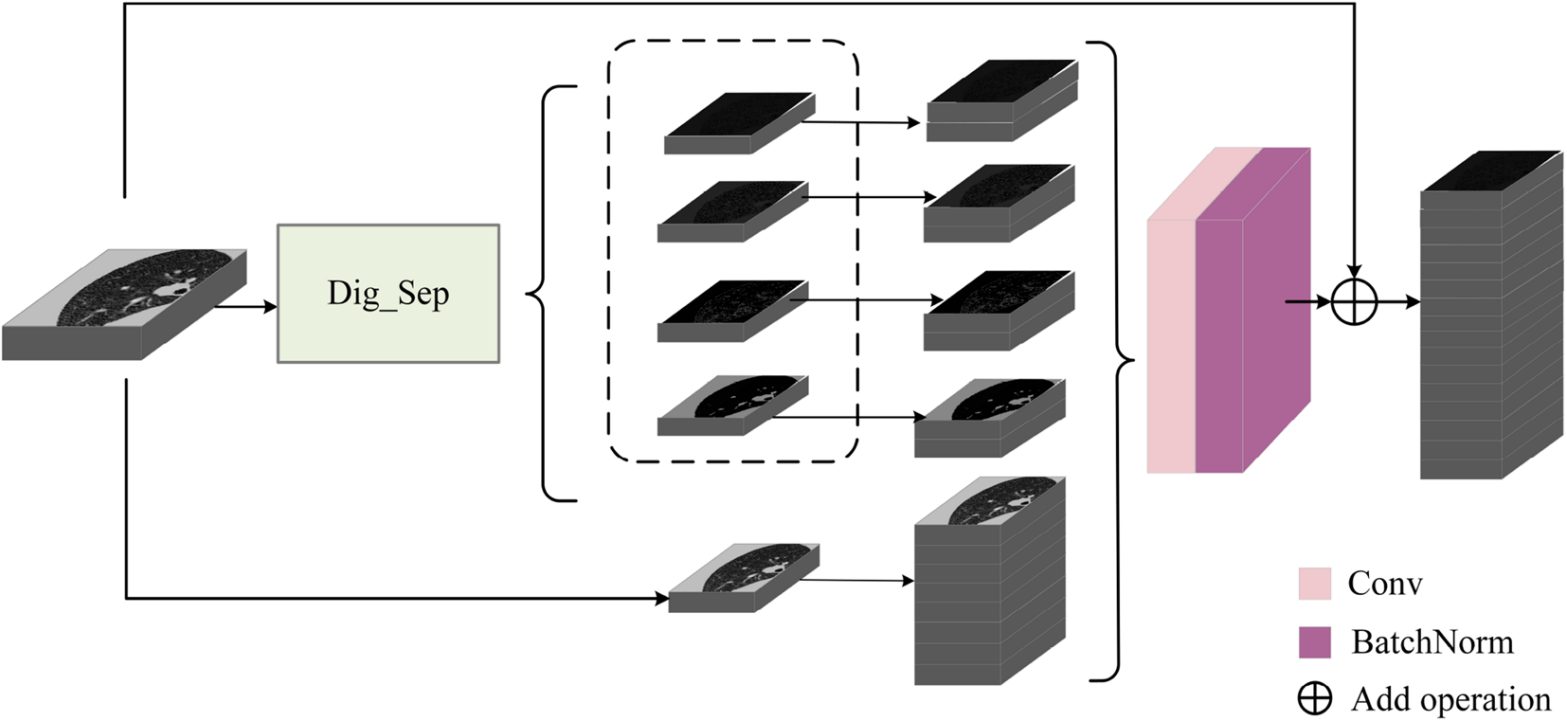
Input model.

First, in the input module of Dig-CS-VNet, we use the Dig_Sep module to separate the input original feature map into four feature maps with different thresholds. Next, the four feature maps are replicated once in the channel dimension to double the final feature map tensor in the channel dimension. In the amplified feature map tensor, except for the four newly added feature maps, the remaining channels are still filled with the input original feature maps. This processing method aims to make full use of the information extracted from the feature maps under different thresholds, improve the network’s perception ability of features at different semantic levels, and thus improve the segmentation performance of the network. The improved input module processing is shown in Fig. 4:

### 3D-CBAM attention mechanism

Convolutional Block Attention Module (CBAM)^39^ is an attention mechanism for enhancing convolutional neural networks. This attention mechanism consists of two modules: the channel attention module(CAM) and the spatial attention module(SAM).

In the CAM process, we first aggregate the spatial information of input features using average-pooling and max-pooling operations, generating two distinct spatial feature maps. Subsequently, with the aid of fully connected layers, we perform channel dimension reduction and expansion on these two sets of feature maps, followed by an element-wise summation operation. Finally, after a sigmoid activation operation, the ultimate channel attention feature is produced. The weights learned through this process are used to weigh the features of each channel, enhancing attention to important channels and reducing attention to less important ones. This contributes to the model’s improved capture of crucial information from different channels in the input images.

In the SAM stage, the output from the CAM phase serves as input features. Initially, global max pooling and global average pooling operations are applied along the channel dimension of the input feature map, resulting in two feature maps with dimensions D$\times$H$\times$W$\times$1. Subsequently, these two feature maps are concatenated along the channel dimension. Following this, a 3$\times$3$\times$3 convolution operation is employed to reduce the channel dimension to 1, yielding a tensor of size D$\times$H$\times$W$\times$1. Finally, the sigmoid activation function is applied to generate the spatial attention feature. SAM utilizes the spatial relationships between elements to generate spatial attention maps, emphasizing spatial positional information on the feature map and complementing channel attention.

**Figure 5:**
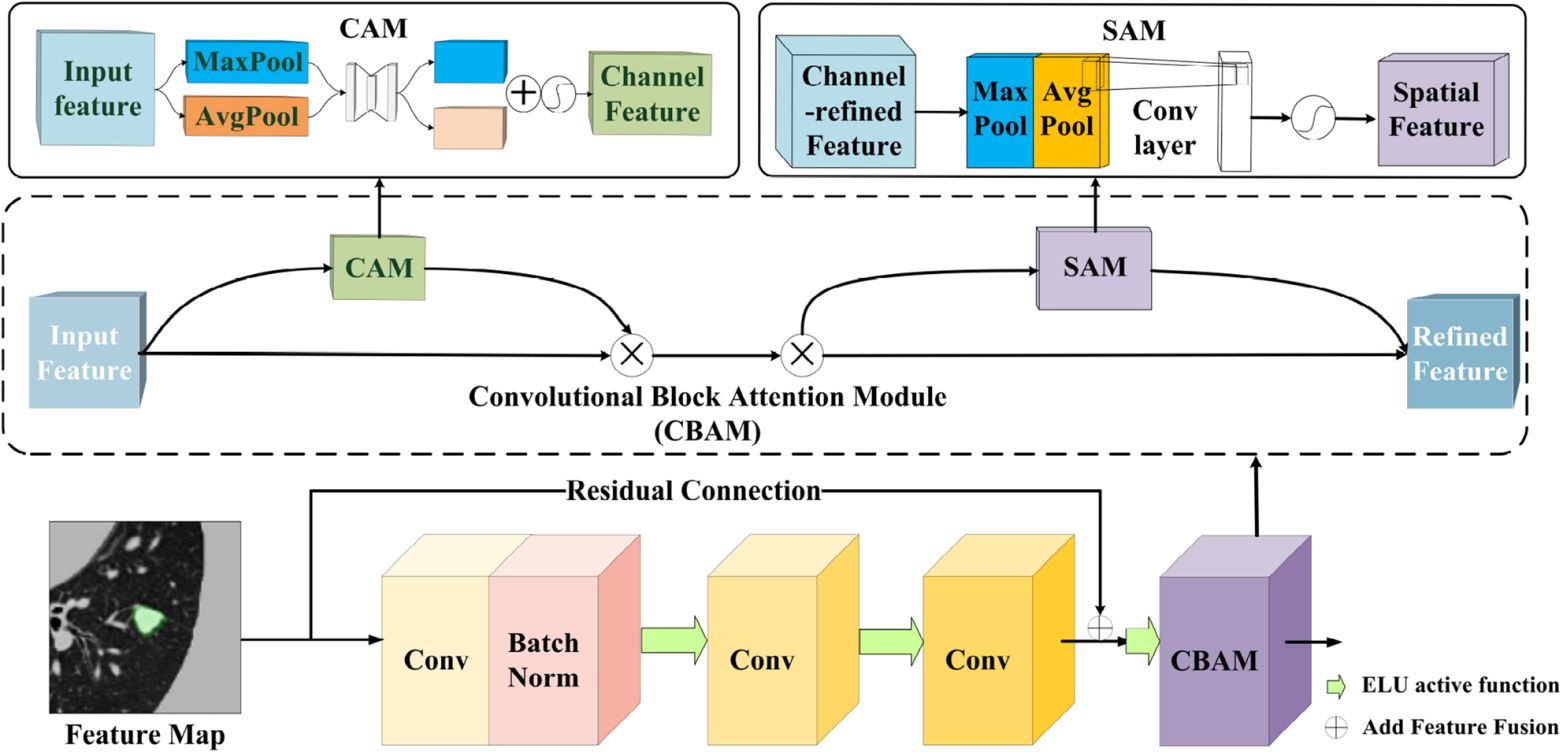
Sampling layer fusion 3D-CBAM attention mechanism.

In lung CT images, the distribution of nodules in the lung parenchyma is relatively random. It has a small proportion, which leads to the problem of having too many feature maps when convolutional neural networks process such images. And most of these feature maps are node-independent and contain a lot of background information, which increases the computational and storage costs of the model and reduces the model’s accuracy and efficiency. Therefore, to solve this problem, we introduce the 3D-CBAM Attention Mechanism to minimize attention to many background regions while paying more attention to small nodule areas in the input image. The downsampling layer incorporating the 3D-CBAM attention mechanism is shown in Fig. 5.

To accommodate the task of segmenting 3D network models, we modify the 2D convolutional kernel of the CBAM attention mechanism into a 3D convolutional kernel and combined with its characteristics, it is placed at the end of each subsampling module, and these attention graphs are multiplied with input feature graphs to carry out adaptive feature refinement, to enhance the network model’s retention of low semantic information and the ability to express specific features in the subsampling process.

### ELU activation function

Exponential Linear Unit (ELU)^40^ is an activation function. Because it can accelerate learning and improve classification accuracy, it is widely used in deep neural networks. Unlike other activation functions, ELU has a negative value, which helps to push the average unit activation near zero, thus accelerating learning. Specifically, when the input is positive, ELU is the same as ReLU; When the information is negative, ELU uses the exponential function to produce a small negative value. This nonlinear form of negative value can avoid the gradient vanishing problem and better handle negative input. This way, the gradient disappearance problem can be avoided, and the convergence can be faster during training.

1 $$\begin{aligned} \qquad \qquad \qquad \qquad \qquad \qquad \qquad ELU=\left\{ \begin{matrix} x &{}, if\quad x>0 \\ \alpha \left( e^{x} -1 \right) &{}, if\quad x\le 0 \end{matrix}\right. \end{aligned}$$

## Experimentations

### Experimental dataset

#### Datasets

- LUNA16 dataset: LUNA16 (Lung Nodule Analysis 2016) is a lung nodule analysis dataset, mainly used for medical image processing and computer-aided diagnosis research. This dataset comes from LIDC-IDRI (Lung Image Database Consortium and Image Database Resource Initiative), which is a large database containing lung computed tomography (CT scan) images. The LUNA16 dataset is extracted from LIDC-IDRI and focuses on the detection and segmentation of pulmonary nodules. The dataset contains CT image data of 888 cases in total^41^.
- LNDb dataset: The LNDb dataset was collected by the São João Hospital in Porto, Portugal (CHUSJ) between 2016 and 2018. The dataset contains a total of 294 CT scans and is used for research on the pulmonary nodule identification and diagnosis system^42^.

#### Image preprocessing

**Figure 6:**
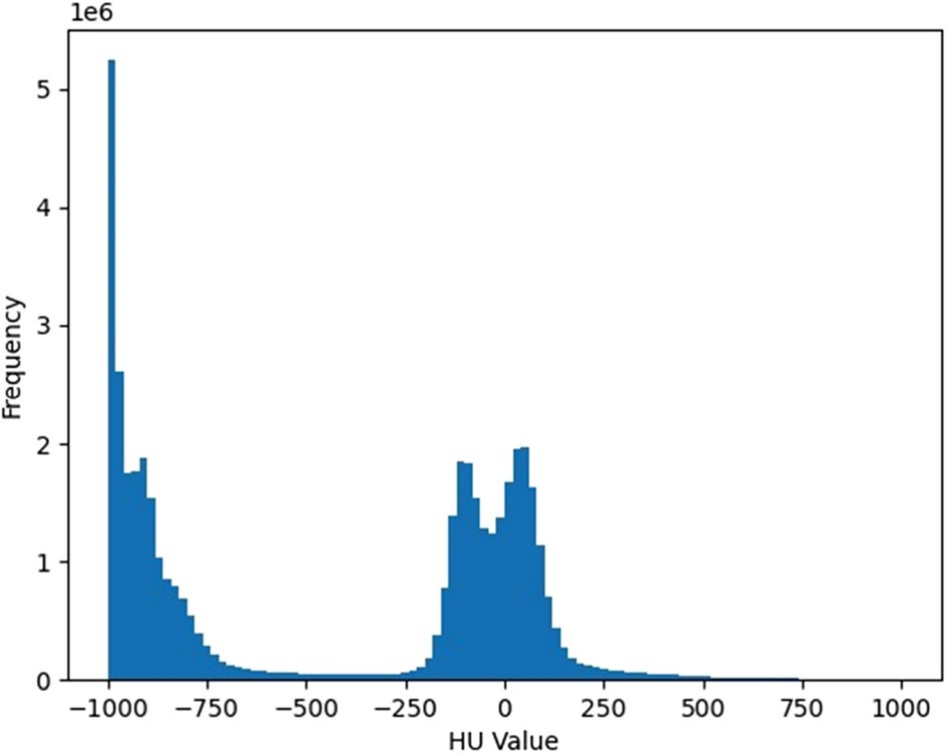
Histogram of CT values distribution for a case.

In the pre-processing stage of the dataset, the histogram of CT values is first drawn according to the CT sample of cases (as shown in Fig. 6), and the distribution range of CT values in the histogram is observed. The content of CT values in the input image is screened and cut to between -1000Hu and 400Hu to remove irrelevant information and noise, such as air and water. Next, the mask data of the lung parenchymal region provided in the dataset was used to further eliminate the areas outside the lungs in the CT image to preserve the lung parenchymal region accurately.

**Figure 7:**
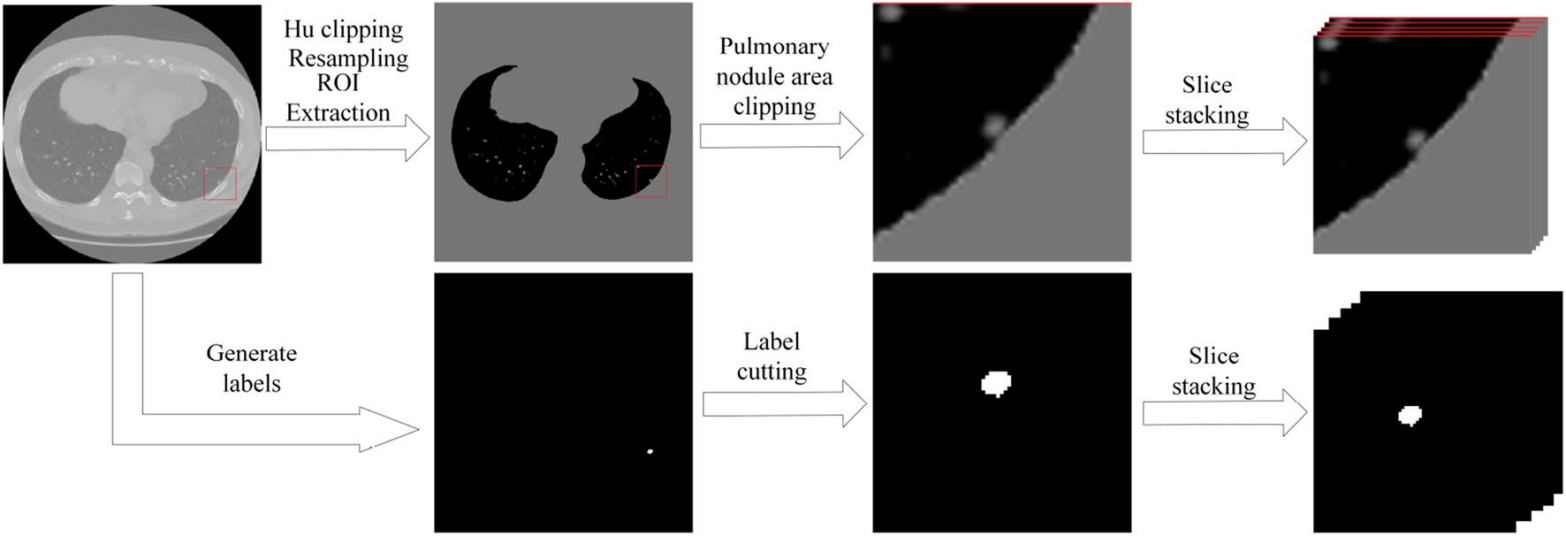
Data preprocessing.

Secondly, the corresponding case label file image (3D) was drawn and generated according to the nodule contour coordinates stored in the annotation file in the case folder (Supplementary Information 1). Because lung nodules occupy a small proportion compared with the whole image layer, data imbalance will occur in the subsequent process, hurting the process and the effect of network training. Therefore, according to the coordinates and maximum diameters of the nodule central site provided in the annotation file, the original image and its corresponding label image were cut into a cube with the size of 96*96*16 pixels to retain the information of a single nodule ultimately. Where 96*96 is the size of the single-layer image, and 16 is the number of image layers contained. Figure 7 shows the preprocessing effect of original image slices and corresponding label files.

#### Data augmentation

**Figure 8:**
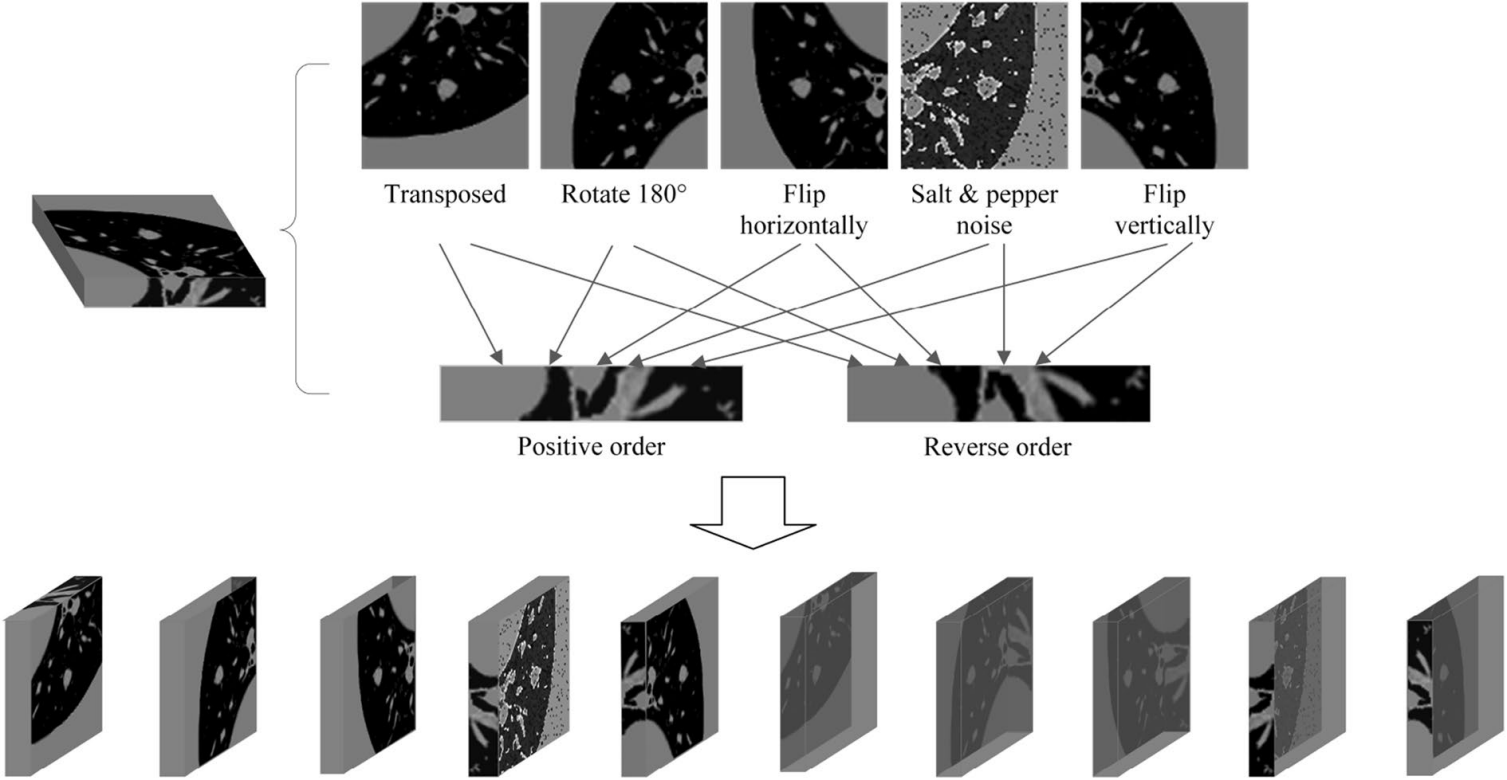
Cross-sectional data augmentation effect image.

Data augmentation is essential in preventing model overfitting, enhancing generalization ability, improving segmentation quality, and enhancing model robustness^43^. In this paper, in addition to conventional rotation and transpose methods, the reverse arrangement and mirror transformation of layers in the Z-axis direction is also carried out for the semantic information of data samples in at least three dimensions. Part of the data used in this paper to augment the cross-sectional effect is shown in Fig. 8. Various data augmentation methods expanded the two datasets to 8746 and 2968 nodule cubes, respectively.

### Loss function and optimization strategy

In deep learning, selecting the appropriate loss function and optimization strategy for the model is very important. Dice Loss was proposed and used for training in the V-Net network model. However, this function would cause profound shock when training samples with unbalanced categories. Therefore, the Cross-Entropy Loss function is used in this paper to measure the gap between model prediction results and accurate labels. The Cross-Entropy Loss function can effectively reduce the classification error and accelerate the convergence rate of the model in the training process. The function expression is as follows:

2 $$\begin{aligned} \qquad \qquad \qquad \qquad \qquad \qquad \qquad \qquad L_{CE} =-\frac{1}{N} {\textstyle \sum _{i=1}^{N}} {\textstyle \sum _{j=1}^{C}}y_{ij}log(p_{ij} ) \end{aligned}$$

where *N* denotes the number of samples, *C* denotes the number of categories, $y_{ij}$ denotes whether the *j*th category of the *i*th sample is the true category (0 or 1), and $p_{ij}$ denotes the probability that the *i*th sample is predicted to be the *j*th category. This loss function implies that the closer the probability of each category the model indicates to the true label, the smaller the loss.

In addition, the Adam optimization strategy^44^ is used to update model parameters, which is an adaptive learning rate optimization algorithm that can dynamically adjust the learning rate to optimize the model’s training. Compared with traditional stochastic gradient descent optimization algorithms, the Adam algorithm can converge faster and avoid falling into the local optimal solution.

### Model training

The hardware environment used in this experiment is: the CPU model is Intel(R) Xeon(R) Platinum 8255C, the GPU model is RTX A5000*2, and the memory is 86G. The software environment is: the operating system is Ubuntu 18.04; the development environment is Anaconda3; code is compiled using Pycharm, and Python3.8 is used as the programming language; the deep learning framework and version are Pytorch-1.7.0, and the GPU driver version is CUDA-11.0.

**Figure 9:**
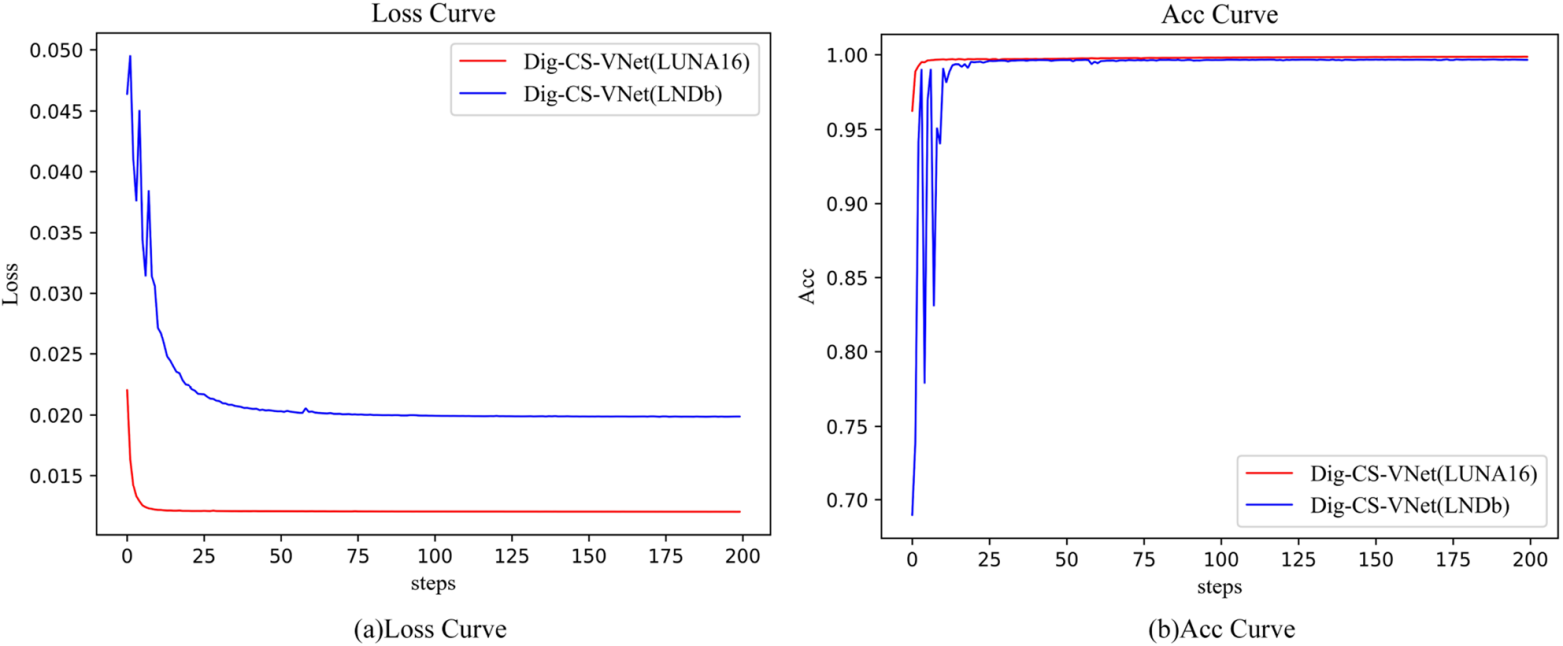
Loss function descent curve and accuracy curve.

We divided the dataset into 10 parts to verify the model’s accuracy. We adopted a ten-fold cross-validation method, of which 9 parts were used as training data and 1 part was used as test data for experiments. The initial learning rate init_lr was set to 0.001, and the Adam stochastic optimization algorithm was used. The network batch size was set to 16. During the training process, when the network model’s loss declined, and each indicator’s trend changed slowly or stagnated, the optimal parameters were saved, and the training process was manually terminated. The learning rate was adjusted to 0.1 times the initial value, and the optimal parameters were loaded to continue the training for 300 iterations. The loss function decline curve and accuracy curve are shown in Fig. 9.

### Evaluation indicators

In this paper, four metrics are used to evaluate the model performance, namely the *DSC* (Dice Similarity Coefficient), *IoU* (Intersection over Union), *SEN* (Sensitivity), and *PPV* (Positive Predictive Value). The *DSC* is mainly used to evaluate the proportion of overlapping parts between the segmentation results predicted by the model and the actual segmentation results. The calculation formulas are as follows :

3 $$\begin{aligned} \qquad \qquad \qquad \qquad \qquad \qquad \qquad \qquad Dice = \frac{{2TP}}{{2TP + FP + FN}} \end{aligned}$$

*IoU* represents the proportion of the intersection of the prediction result and the accurate label to the union of the prediction result and the precise label. The calculation formulas are as follows :

4 $$\begin{aligned} \qquad \qquad \qquad \qquad \qquad \qquad \qquad \qquad IoU = \frac{{TP}}{{TP + FP + FN}} \end{aligned}$$

*SEN* represents the proportion of the actual positive samples that the model correctly predicts as positive samples. The calculation formula is :

5 $$\begin{aligned} \qquad \qquad \qquad \qquad \qquad \qquad \qquad \qquad SEN = \frac{{TP}}{{TP + FN}} \end{aligned}$$

*PPV* represents the proportion of positive samples predicted by the model and the actual positive samples. The calculation formulas are as follows :

6 $$\begin{aligned} \qquad \qquad \qquad \qquad \qquad \qquad \qquad \qquad PPV = \frac{{TP}}{{TP + FP}} \end{aligned}$$

In the formula, *TP* is the number of true positives, *FP* is the number of false positives, and *FN* is the number of false negatives. The above four evaluation indicators are between 0 and 1. The higher the segmentation accuracy of the network model, the closer the value of each indicator is to 1.

### Experimental analysis

#### Ablation experiments

1. Improved Model Ablation Experiments

To verify the effectiveness of each part of the method in this paper, four ablation experiments are done with V-Net as the base network to prove the effectiveness of each part of the improvement work. The results of various network segmentation indicators in ablation experiments are shown in Table 1.

**Table 1:** Comparison of ablation experiments on each model.

**Models**	**IoU (%)**	**DSC (%)**	**SEN (%)**	**PPV (%)**
V-Net	83.9	91.2	88.5	94.1
V-Net+ELU	85.5	92.5	89.7	94.8
V-Net+ELU+Dig_Sep	89.1	94.2	91.9	96.8
V-Net+ELU+3D-CBAM	88.8	94	91.1	97.2
Dig-CS-VNet	90.3	94.9	92.7	97.2

**Figure 10:**
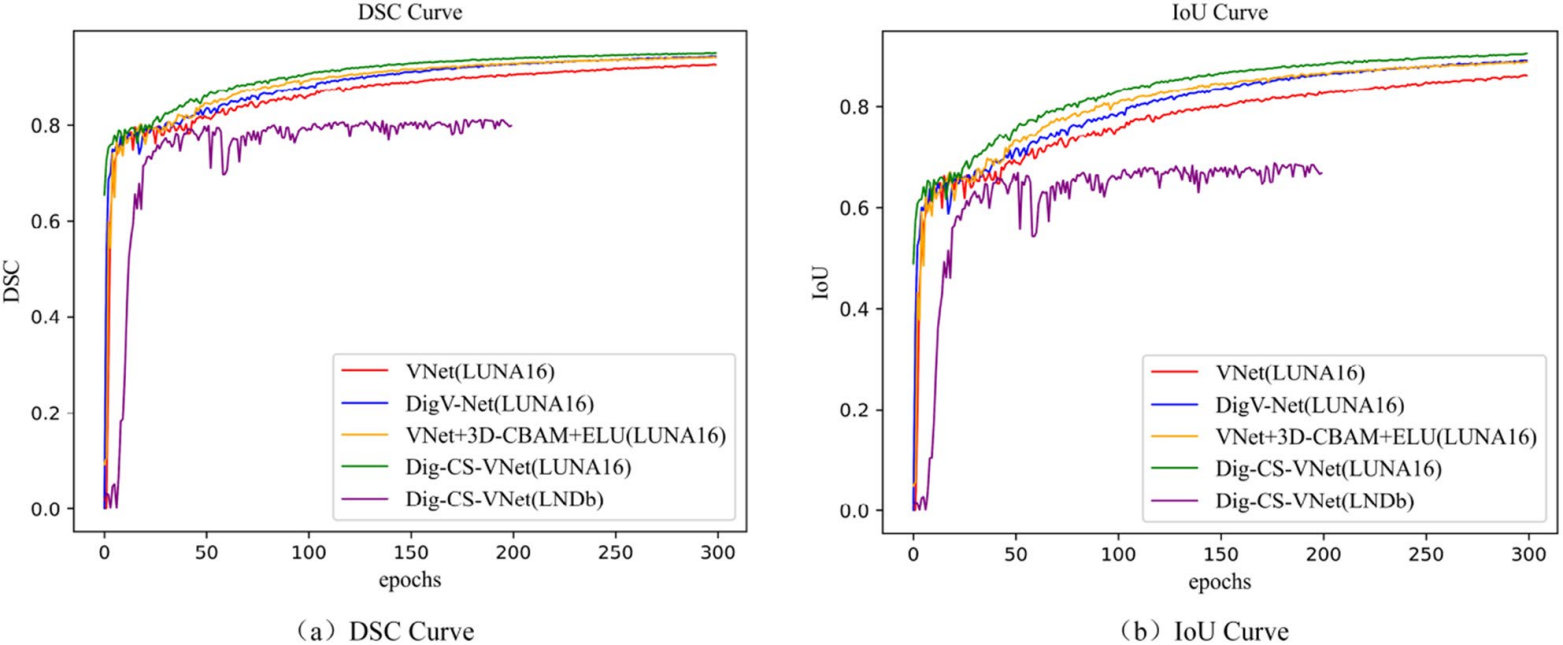
DSC and IoU accuracy variation curves.

Compared with the benchmark network model, V-Net+ELU improves the DSC coefficient by 1.3 percentage points compared to V-Net. The DSC coefficient reaches 92.5%. In addition, compared with V-Net + ELU, which only changes the activation function, the improved network with the Dig_Sep module and 3D-CBAM module achieves 94.2% and 94% in the DSC coefficient, respectively. Compared with the PPV index, the attention mechanism is better for optimistic sample prediction than the Dig_Sep module, indicating that the 3D-CBAM attention mechanism enhances specific information’s attention and extraction ability. The final improved network model architecture Dig-CS-VNet shows significant improvement in the proportion of overlapping regions and optimistic sample prediction compared to other network models. The variation curves of IoU and DSC coefficients for each model are shown in Fig. 10.

(2) Data Augmentation Ablation Experiments

To verify the effectiveness of data augmentation, we conducted experiments on the original and augmented datasets using the benchmark network V-Net and the improved model separately and compared the results.At this stage, we conducted a set of experiments on each of the baseline model and the improved model. Each set of experiments includes 3 tests. namely testing on the original dataset without data augmentation; testing on the dataset augmented using traditional methods such as rotation, scaling, translation, and grayscale transformation; testing on the using the dataset augmented by the method proposed in this article. Testing on the augmented dataset. The performance results before and after data augmentation are shown in Tables 2 and 3.

**Table 2:** Comparison of ablation experiments on V-Net.

**V-Net**	**IoU (%)**	**DSC (%)**	**SEN (%)**	**PPV (%)**
Before augmentation	69	81.5	73.7	90.7
Traditional Method	74.4	84.8	81.3	88.9
Ours	83.9	91.2	88.5	94.1

As shown in Table 2, by comparing the experimental indicators of the baseline, the method proposed in this paper is about 9.5 percentage points higher than the traditional method on *IoU* and 6.4 percentage points higher on *DSC*. *SEN* and *PPV* increased by 7.2 and 5.2, respectively. Compared with the unaugmented data, the accuracy of the first four indicators increased by 14.9, 9.7, 14.8 and 3.4 percentage points, respectively. It shows that the V-Net using the data augmentation method proposed in this paper has a higher improvement in the overlap ratio and positive prediction of the segmented region. The traditional data augmentation reduces the *PPV* index by about 1.8%, indicating that the traditional augmentation scheme is not conducive to the prediction of correct samples in the V-Net segmentation process.

**Table 3:** Comparison of ablation experiments on Dig-CS-VNet.

**Dig-CS-VNet**	**IoU (%)**	**DSC (%)**	**SEN (%)**	**PPV (%)**
Before augmentation	72.9	84.6	81.4	88.5
Traditional Method	76.5	86	82.8	89.8
Ours	90.3	94.9	92.7	97.2

By comparison, Dig-CS-VNet using the data augmentation method proposed in this paper in Table 3, compared with the experimental results of the original dataset, *IoU* increased by about 17.4 percentage points, *DSC* coefficient increased by about 10.3 percentage points, *SEN* and *PPV* increased by 11.3% and 8.7% respectively. Compared with the traditional data augmentation method, there is about 13.8 percentage points improvement in *IoU*, 8.9 percentage points improvement in *DSC*, and 9.9% and 7.4% improvement in *SEN* and *PPV*, respectively. Experiments show that the augmentation method proposed in this paper still has a great improvement in improving the network model. And the improved model does not appear *PPV* reduction phenomenon in the process of using the traditional augmentation method, which proves that the improved network has better positive sample prediction ability than V-Net. The above experiments prove that the data augmentation scheme designed in this paper is more effective in improving the segmentation accuracy and generalization ability of the model.

As shown in the table above, during the experiment, the *IoU* changed significantly. After repeated tests and comparisons, we found that the scaling operation was not conducive to improving the accuracy of the model during the research of this article. We tested the dataset without scaling augmentation., the baseline model and the improved model are up to 3.3% and 4.1% higher on *DSC* respectively, and the *IoU* performance is 4.4% and 5.6% higher respectively. In addition, improvements in data augmentation methods and enrichment of data samples are also important reasons for the significant improvement in model performance.

**Figure 11:**
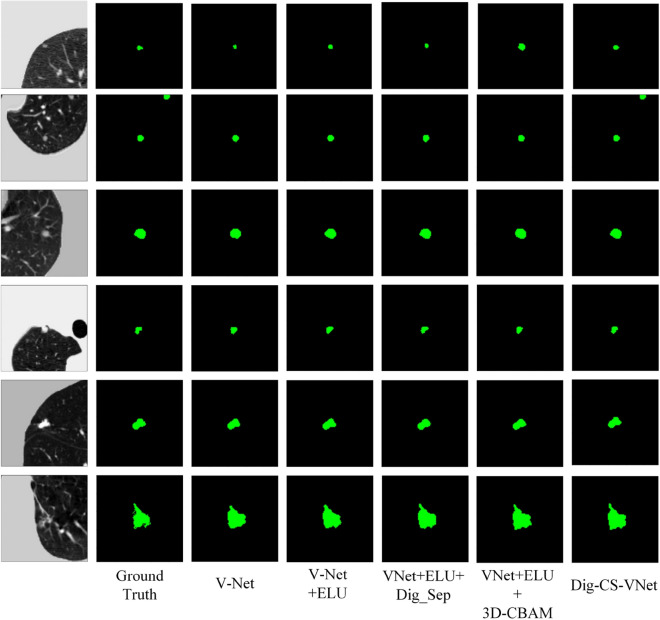
Nodule segmentation result image.

The following Fig. 11 shows some of the nodule segmentation results (Supplementary Information 2) in the improvement stages from the benchmark network model to the complete improvement network. From top to bottom, we found that Dig-CS-VNet has far more advantages than other models for marginal micro nodules. For common solitary pulmonary nodules, the effects of each network are similar, among which V-Net + Dig_Sep and Dig-CS-VNet are almost consistent with Ground Truth (GT), indicating the effectiveness of extracting hierarchical features. In the comparison of the segmentation effect of irregular pulmonary nodules, both small pulmonary nodules and general pulmonary nodules performed best on the completely improved network. Finally, for pulmonary nodules with unclear contours, V-Net + 3D-CBAM and Dig-CS-VNet have the best segmentation effect, and the edge contour is closer to GT, indicating that the attention mechanism has certain advantages for specific semantic information extraction. The segmentation effect verifies the effectiveness of the improved network.

#### Comparison of experimental effects of different algorithms

In this section, the algorithm is evaluated and compared with other algorithms of the same research work using the indicator of *DSC* introduced above.

**Table 4:** Comparison of model segmentation effects.

**Method**	**Dataset**	**DSC(%)**
BBCLstm-Unet^9^	LUNA16	90.83
U-Det^11^	LUNA16	90.83
CTBP-Net^13^	LUNA16	91.68
MDFN^14^	LUNA16	89.19
LNCDS^15^	LNDb	80.0
Multi-Feature Fusion^18^	LUNA16	83.5
SM-RNet^19^	LUNA16	86.5
CSE-GAN^32^	LUNA16	80.74
3D-UNet+CRF^34^	LUNA16	93.25
DAS-Net^45^	LUNA16	92.05
AWEU-Net^46^	LUNA16	90.35
nnUNet+ SaTTCA^47^	LNDb	71.46
Dig-CS-VNet (Ours)	LUNA16/LNDb	94.9/81.1

In order to ensure the consistency of the experiment, Table 4 only compares with the segmentation method under the homologous dataset.The experimental comparison shows that the improved model in this paper performs well on *DSC* indicator, and the performance is more prominent under the U-Net architecture.

## Results

This paper proposes an Dig-CS-VNet model with a V-Net architecture for the lung nodule segmentation, which improved based on pixel threshold separation and attention mechanism. This model can effectively solve the problem that the small sample size of the medical dataset could be more conducive to training on the one hand. On the other hand, this model can utilize hierarchical feature information and detail feature information to overcome resulting in poor robustness and inaccurate detail segmentation. The experimental results on the public datasets LUNA16 and LNDb demonstrate that our method has achieved significant segmentation accuracy. Specifically, the segmentation metrics, including the *DSC* and *SEN*, reached high precision at 94.9% and 81.1%, respectively. Furthermore, our method exhibited performance with sensitivity at 92.7% and 76.9%, respectively. These results surpass the majority of existing segmentation methods, highlighting the outstanding performance of our approach.

### Supplementary Information

Supplementary Information 1.

Supplementary Information 2.

## Data Availability

The datasets utilized and/or analyzed during the current study are accessible via their respective official designated websites. The LUNA16 dataset can be obtained from Website1 (https://zenodo.org/records/3723295) and Website2 (https://zenodo.org/records/4121926). Similarly, the LNdb dataset is accessible from Website3 (https://zenodo.org/records/6613714). For additional details about both datasets, please refer to the official websites of the LUng Nodule Analysis 2016 (https://luna16.grand-challenge.org/) and the Grand Challenge on Automatic Lung Cancer Patient Management (https://lndb.grand-challenge.org/). In addition, other data information generated during the experiment will be collated and stored in the Luna-16-nodules-volume repository (https://github.com/DigCS/Luna-16-nodules-volume). The datasets generated during and/or analysed during the current study are available from the corresponding author on reasonable request.
