# Supplementary figures and images for: An improved V-Net lung nodule segmentation model based on pixel threshold separation and attention mechanism (part 2 of 2)
Source: Sci Rep. 2024 Feb 27;14:4743. doi: 10.1038/s41598-024-55178-3 (PMC10899216; doi:10.1038/s41598-024-55178-3)

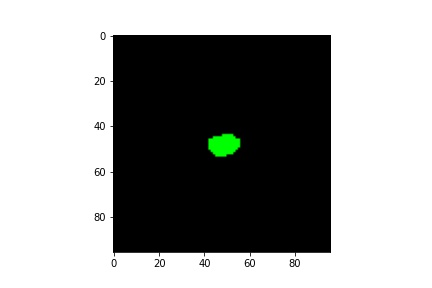

Supplement: Supplementary file 2 — Supplementary Information 2. [file 41598_2024_55178_MOESM2_ESM.zip › dcvnet/13_0_7.jpg]

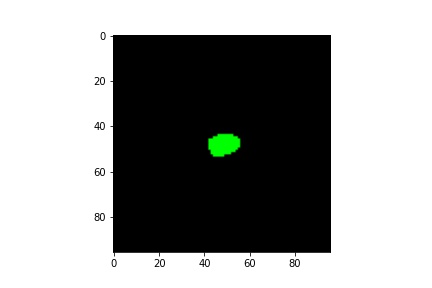

Supplement: Supplementary file 2 — Supplementary Information 2. [file 41598_2024_55178_MOESM2_ESM.zip › dcvnet/13_0_8.jpg]

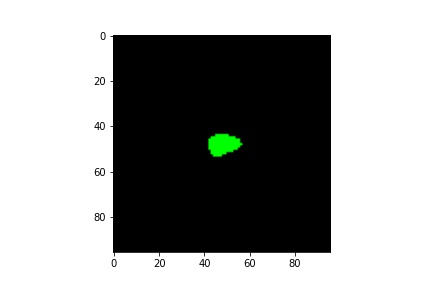

Supplement: Supplementary file 2 — Supplementary Information 2. [file 41598_2024_55178_MOESM2_ESM.zip › dcvnet/13_0_9.jpg]

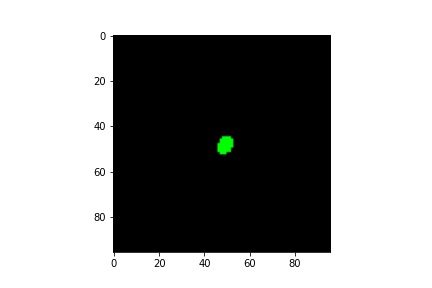

Supplement: Supplementary file 2 — Supplementary Information 2. [file 41598_2024_55178_MOESM2_ESM.zip › dcvnet/13_1_0.jpg]

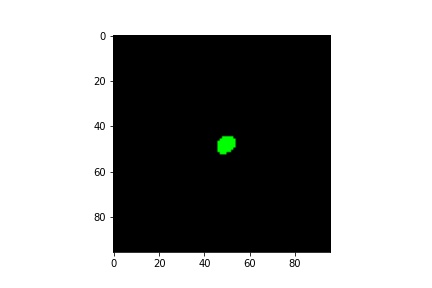

Supplement: Supplementary file 2 — Supplementary Information 2. [file 41598_2024_55178_MOESM2_ESM.zip › dcvnet/13_1_1.jpg]

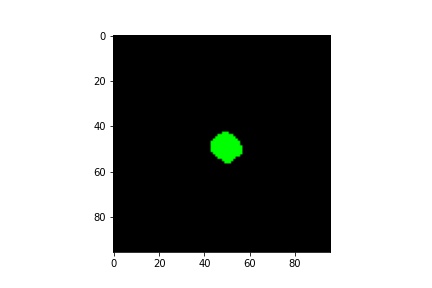

Supplement: Supplementary file 2 — Supplementary Information 2. [file 41598_2024_55178_MOESM2_ESM.zip › dcvnet/13_1_10.jpg]

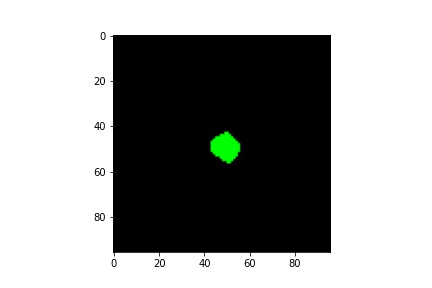

Supplement: Supplementary file 2 — Supplementary Information 2. [file 41598_2024_55178_MOESM2_ESM.zip › dcvnet/13_1_11.jpg]

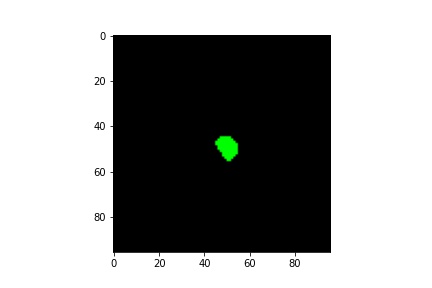

Supplement: Supplementary file 2 — Supplementary Information 2. [file 41598_2024_55178_MOESM2_ESM.zip › dcvnet/13_1_12.jpg]

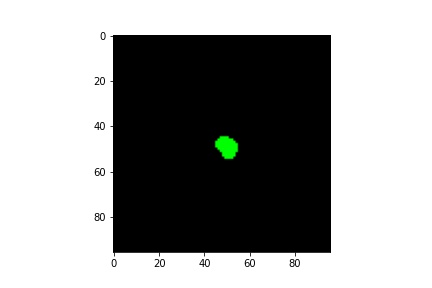

Supplement: Supplementary file 2 — Supplementary Information 2. [file 41598_2024_55178_MOESM2_ESM.zip › dcvnet/13_1_13.jpg]

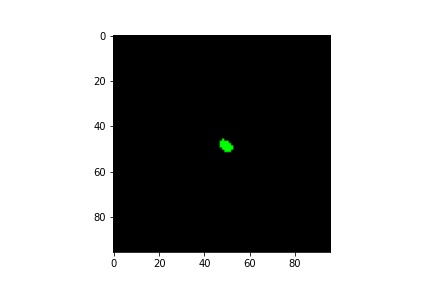

Supplement: Supplementary file 2 — Supplementary Information 2. [file 41598_2024_55178_MOESM2_ESM.zip › dcvnet/13_1_14.jpg]

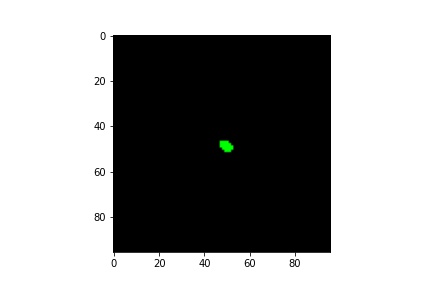

Supplement: Supplementary file 2 — Supplementary Information 2. [file 41598_2024_55178_MOESM2_ESM.zip › dcvnet/13_1_15.jpg]

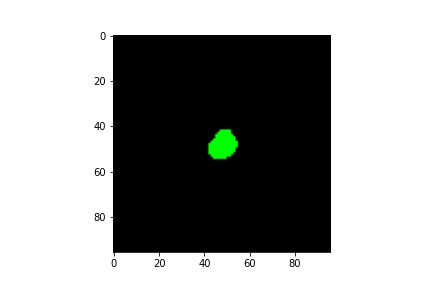

Supplement: Supplementary file 2 — Supplementary Information 2. [file 41598_2024_55178_MOESM2_ESM.zip › dcvnet/13_1_2.jpg]

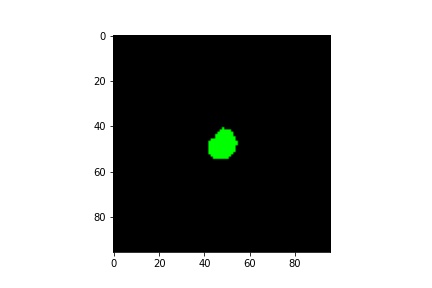

Supplement: Supplementary file 2 — Supplementary Information 2. [file 41598_2024_55178_MOESM2_ESM.zip › dcvnet/13_1_3.jpg]

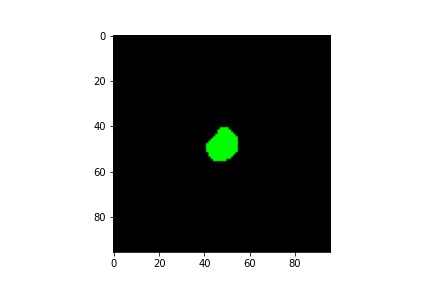

Supplement: Supplementary file 2 — Supplementary Information 2. [file 41598_2024_55178_MOESM2_ESM.zip › dcvnet/13_1_4.jpg]

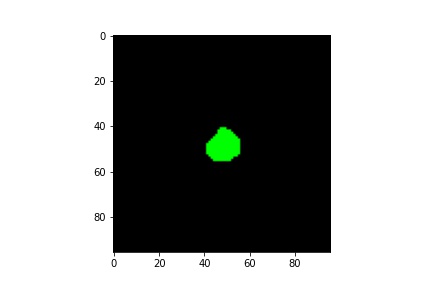

Supplement: Supplementary file 2 — Supplementary Information 2. [file 41598_2024_55178_MOESM2_ESM.zip › dcvnet/13_1_5.jpg]

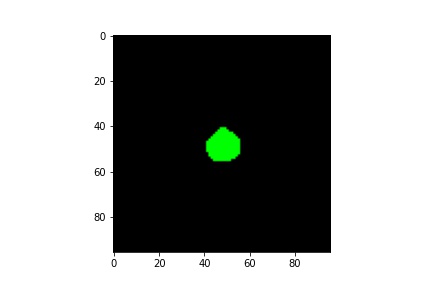

Supplement: Supplementary file 2 — Supplementary Information 2. [file 41598_2024_55178_MOESM2_ESM.zip › dcvnet/13_1_6.jpg]

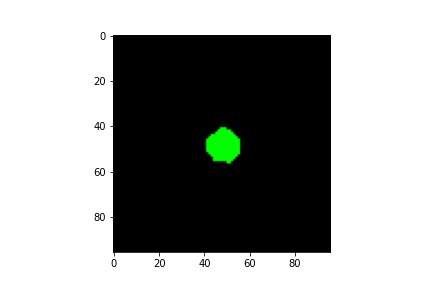

Supplement: Supplementary file 2 — Supplementary Information 2. [file 41598_2024_55178_MOESM2_ESM.zip › dcvnet/13_1_7.jpg]

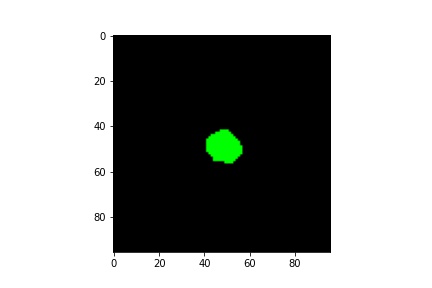

Supplement: Supplementary file 2 — Supplementary Information 2. [file 41598_2024_55178_MOESM2_ESM.zip › dcvnet/13_1_8.jpg]

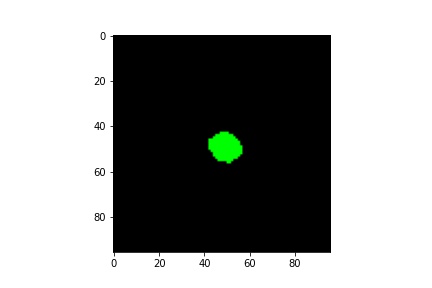

Supplement: Supplementary file 2 — Supplementary Information 2. [file 41598_2024_55178_MOESM2_ESM.zip › dcvnet/13_1_9.jpg]

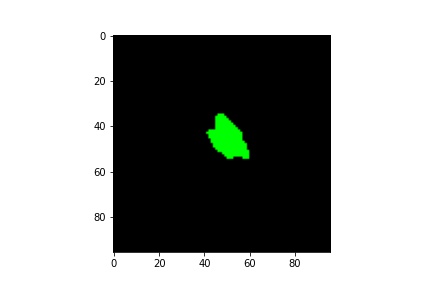

Supplement: Supplementary file 2 — Supplementary Information 2. [file 41598_2024_55178_MOESM2_ESM.zip › dcvnet/14_0_0.jpg]

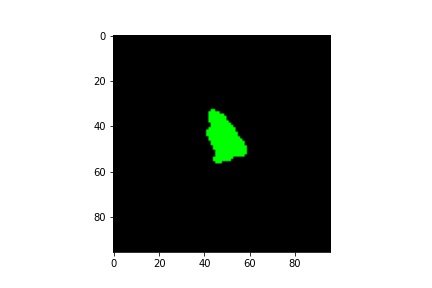

Supplement: Supplementary file 2 — Supplementary Information 2. [file 41598_2024_55178_MOESM2_ESM.zip › dcvnet/14_0_1.jpg]

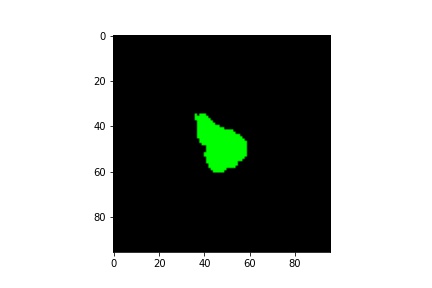

Supplement: Supplementary file 2 — Supplementary Information 2. [file 41598_2024_55178_MOESM2_ESM.zip › dcvnet/14_0_10.jpg]

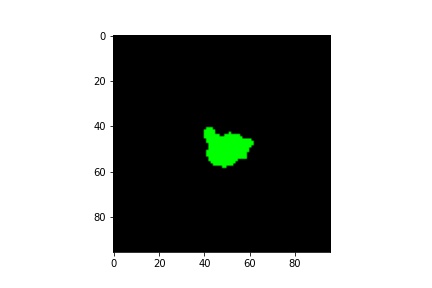

Supplement: Supplementary file 2 — Supplementary Information 2. [file 41598_2024_55178_MOESM2_ESM.zip › dcvnet/14_0_11.jpg]

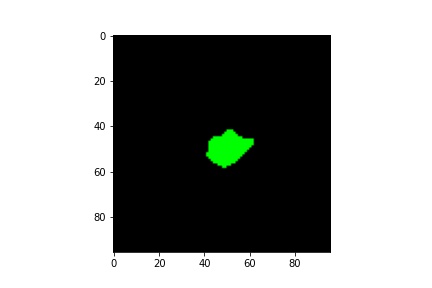

Supplement: Supplementary file 2 — Supplementary Information 2. [file 41598_2024_55178_MOESM2_ESM.zip › dcvnet/14_0_12.jpg]

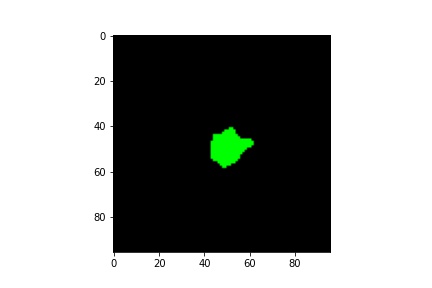

Supplement: Supplementary file 2 — Supplementary Information 2. [file 41598_2024_55178_MOESM2_ESM.zip › dcvnet/14_0_13.jpg]

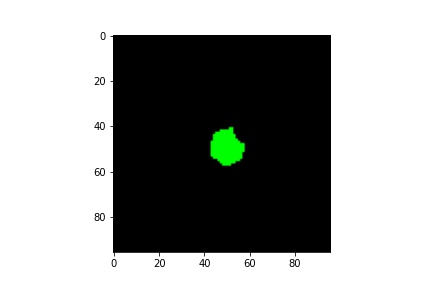

Supplement: Supplementary file 2 — Supplementary Information 2. [file 41598_2024_55178_MOESM2_ESM.zip › dcvnet/14_0_14.jpg]

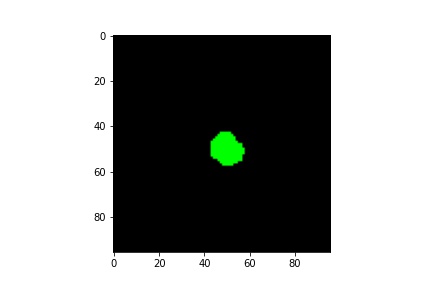

Supplement: Supplementary file 2 — Supplementary Information 2. [file 41598_2024_55178_MOESM2_ESM.zip › dcvnet/14_0_15.jpg]

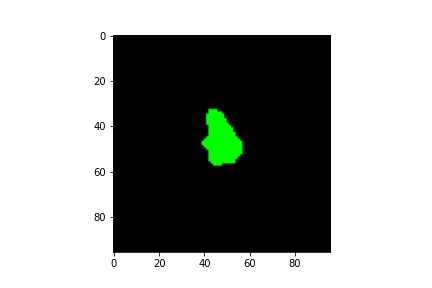

Supplement: Supplementary file 2 — Supplementary Information 2. [file 41598_2024_55178_MOESM2_ESM.zip › dcvnet/14_0_2.jpg]

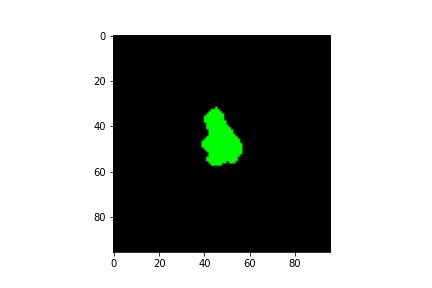

Supplement: Supplementary file 2 — Supplementary Information 2. [file 41598_2024_55178_MOESM2_ESM.zip › dcvnet/14_0_3.jpg]

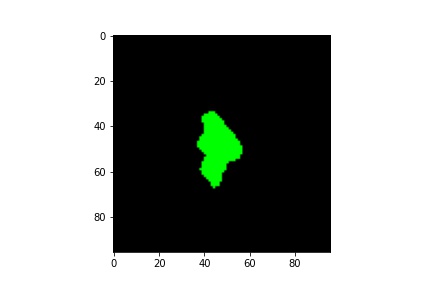

Supplement: Supplementary file 2 — Supplementary Information 2. [file 41598_2024_55178_MOESM2_ESM.zip › dcvnet/14_0_4.jpg]

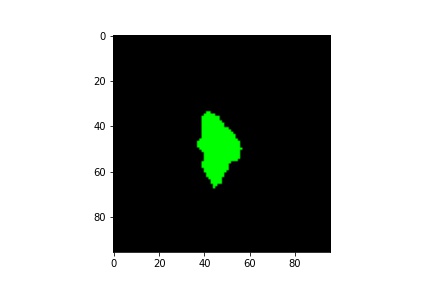

Supplement: Supplementary file 2 — Supplementary Information 2. [file 41598_2024_55178_MOESM2_ESM.zip › dcvnet/14_0_5.jpg]

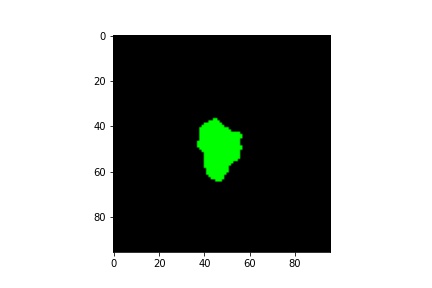

Supplement: Supplementary file 2 — Supplementary Information 2. [file 41598_2024_55178_MOESM2_ESM.zip › dcvnet/14_0_6.jpg]

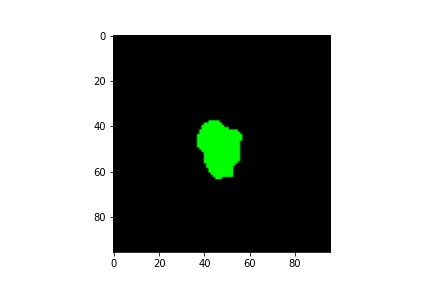

Supplement: Supplementary file 2 — Supplementary Information 2. [file 41598_2024_55178_MOESM2_ESM.zip › dcvnet/14_0_7.jpg]

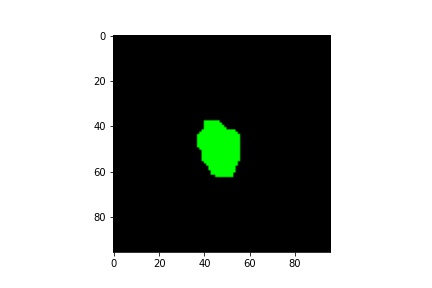

Supplement: Supplementary file 2 — Supplementary Information 2. [file 41598_2024_55178_MOESM2_ESM.zip › dcvnet/14_0_8.jpg]

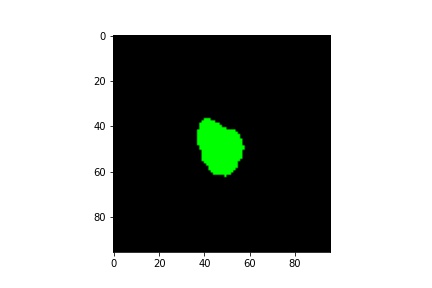

Supplement: Supplementary file 2 — Supplementary Information 2. [file 41598_2024_55178_MOESM2_ESM.zip › dcvnet/14_0_9.jpg]

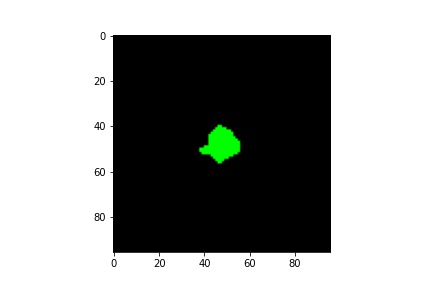

Supplement: Supplementary file 2 — Supplementary Information 2. [file 41598_2024_55178_MOESM2_ESM.zip › dcvnet/14_1_0.jpg]

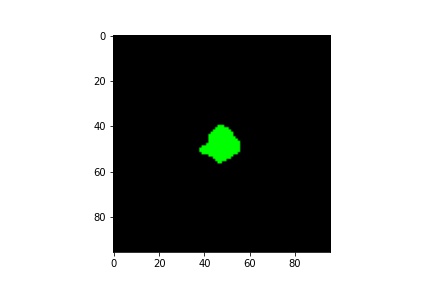

Supplement: Supplementary file 2 — Supplementary Information 2. [file 41598_2024_55178_MOESM2_ESM.zip › dcvnet/14_1_1.jpg]

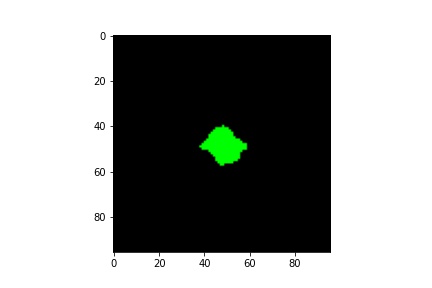

Supplement: Supplementary file 2 — Supplementary Information 2. [file 41598_2024_55178_MOESM2_ESM.zip › dcvnet/14_1_10.jpg]

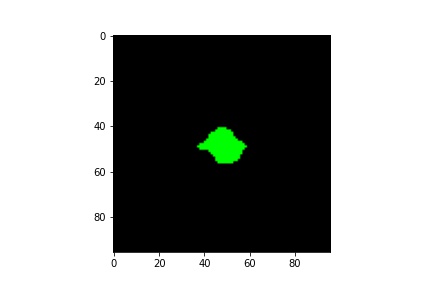

Supplement: Supplementary file 2 — Supplementary Information 2. [file 41598_2024_55178_MOESM2_ESM.zip › dcvnet/14_1_11.jpg]

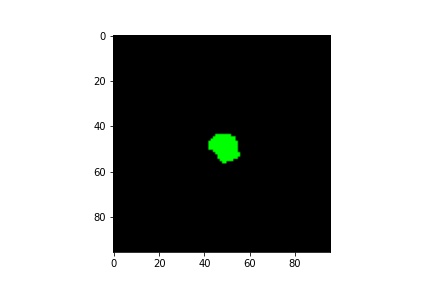

Supplement: Supplementary file 2 — Supplementary Information 2. [file 41598_2024_55178_MOESM2_ESM.zip › dcvnet/14_1_12.jpg]

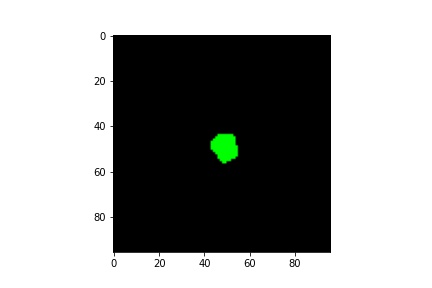

Supplement: Supplementary file 2 — Supplementary Information 2. [file 41598_2024_55178_MOESM2_ESM.zip › dcvnet/14_1_13.jpg]

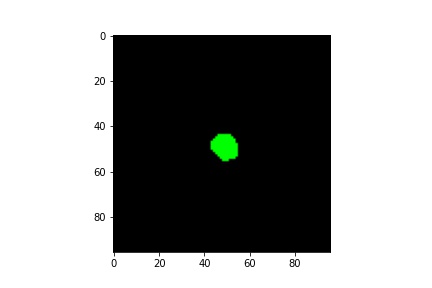

Supplement: Supplementary file 2 — Supplementary Information 2. [file 41598_2024_55178_MOESM2_ESM.zip › dcvnet/14_1_14.jpg]

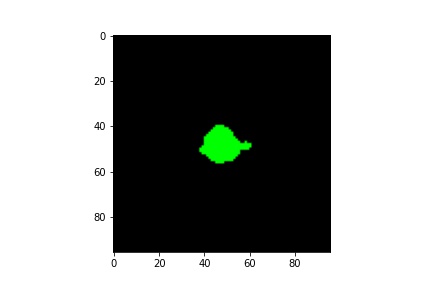

Supplement: Supplementary file 2 — Supplementary Information 2. [file 41598_2024_55178_MOESM2_ESM.zip › dcvnet/14_1_2.jpg]

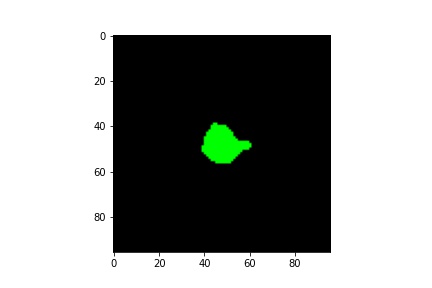

Supplement: Supplementary file 2 — Supplementary Information 2. [file 41598_2024_55178_MOESM2_ESM.zip › dcvnet/14_1_3.jpg]

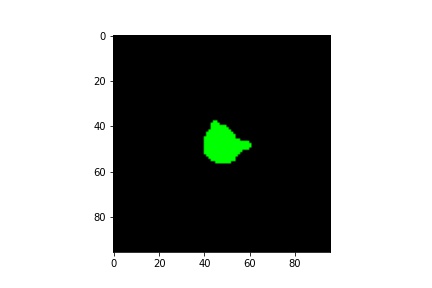

Supplement: Supplementary file 2 — Supplementary Information 2. [file 41598_2024_55178_MOESM2_ESM.zip › dcvnet/14_1_4.jpg]

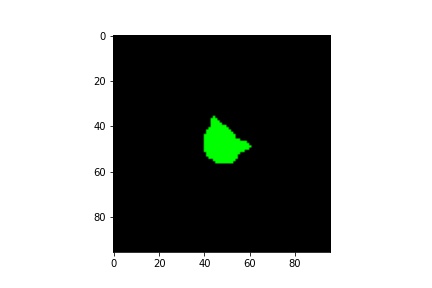

Supplement: Supplementary file 2 — Supplementary Information 2. [file 41598_2024_55178_MOESM2_ESM.zip › dcvnet/14_1_5.jpg]

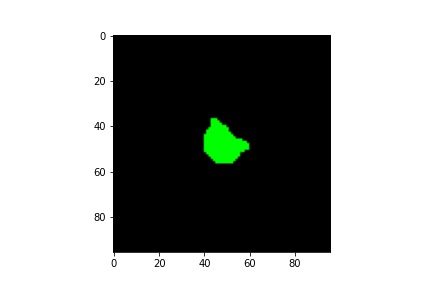

Supplement: Supplementary file 2 — Supplementary Information 2. [file 41598_2024_55178_MOESM2_ESM.zip › dcvnet/14_1_6.jpg]

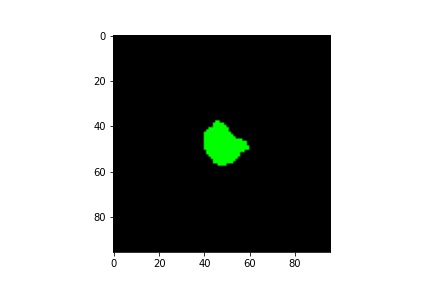

Supplement: Supplementary file 2 — Supplementary Information 2. [file 41598_2024_55178_MOESM2_ESM.zip › dcvnet/14_1_7.jpg]

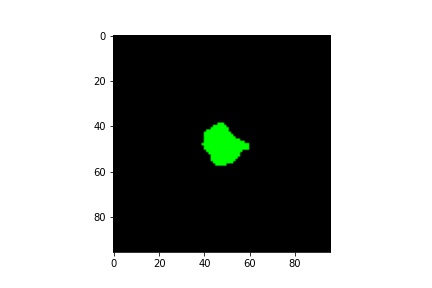

Supplement: Supplementary file 2 — Supplementary Information 2. [file 41598_2024_55178_MOESM2_ESM.zip › dcvnet/14_1_8.jpg]

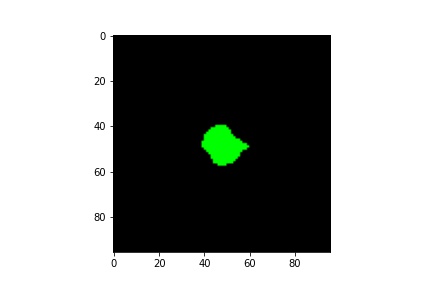

Supplement: Supplementary file 2 — Supplementary Information 2. [file 41598_2024_55178_MOESM2_ESM.zip › dcvnet/14_1_9.jpg]

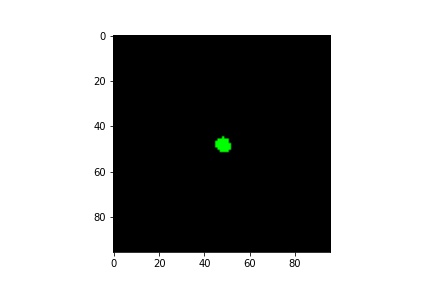

Supplement: Supplementary file 2 — Supplementary Information 2. [file 41598_2024_55178_MOESM2_ESM.zip › dcvnet/15_0_10.jpg]
