# Supplementary figures and images for: An improved V-Net lung nodule segmentation model based on pixel threshold separation and attention mechanism (part 1 of 2)
Source: Sci Rep. 2024 Feb 27;14:4743. doi: 10.1038/s41598-024-55178-3 (PMC10899216; doi:10.1038/s41598-024-55178-3)

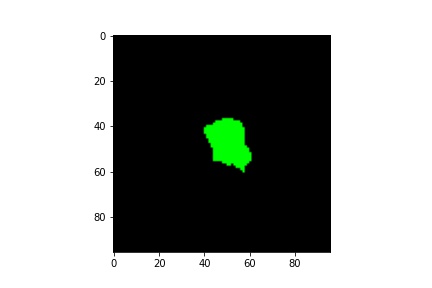

Supplement: Supplementary file 2 — Supplementary Information 2. [file 41598_2024_55178_MOESM2_ESM.zip › dcvnet/0_0_0.jpg]

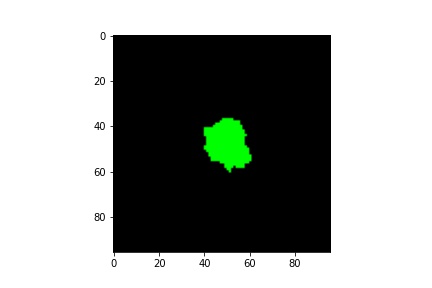

Supplement: Supplementary file 2 — Supplementary Information 2. [file 41598_2024_55178_MOESM2_ESM.zip › dcvnet/0_0_1.jpg]

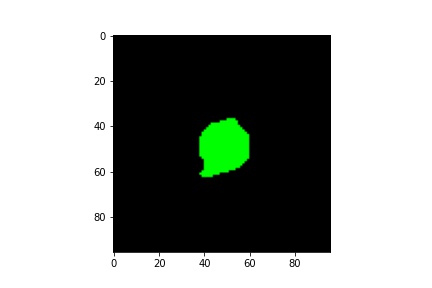

Supplement: Supplementary file 2 — Supplementary Information 2. [file 41598_2024_55178_MOESM2_ESM.zip › dcvnet/0_0_10.jpg]

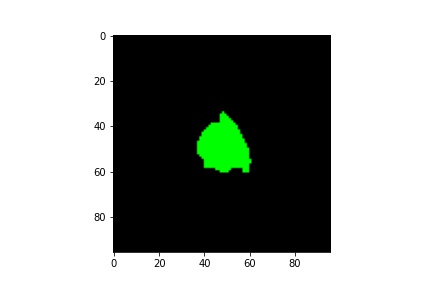

Supplement: Supplementary file 2 — Supplementary Information 2. [file 41598_2024_55178_MOESM2_ESM.zip › dcvnet/0_0_11.jpg]

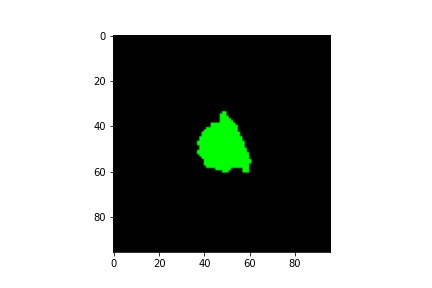

Supplement: Supplementary file 2 — Supplementary Information 2. [file 41598_2024_55178_MOESM2_ESM.zip › dcvnet/0_0_12.jpg]

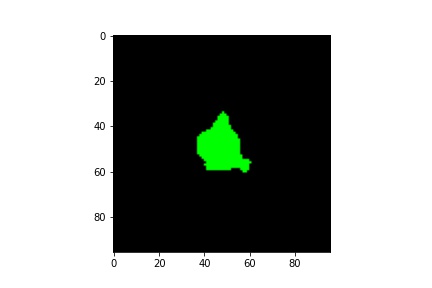

Supplement: Supplementary file 2 — Supplementary Information 2. [file 41598_2024_55178_MOESM2_ESM.zip › dcvnet/0_0_13.jpg]

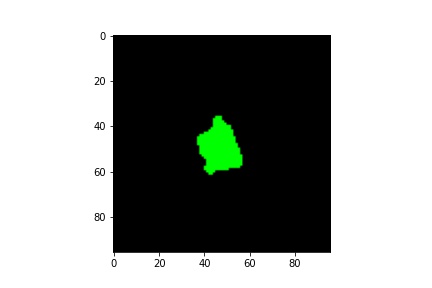

Supplement: Supplementary file 2 — Supplementary Information 2. [file 41598_2024_55178_MOESM2_ESM.zip › dcvnet/0_0_14.jpg]

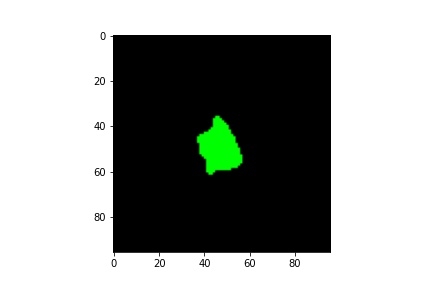

Supplement: Supplementary file 2 — Supplementary Information 2. [file 41598_2024_55178_MOESM2_ESM.zip › dcvnet/0_0_15.jpg]

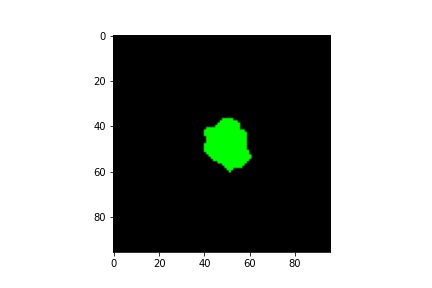

Supplement: Supplementary file 2 — Supplementary Information 2. [file 41598_2024_55178_MOESM2_ESM.zip › dcvnet/0_0_2.jpg]

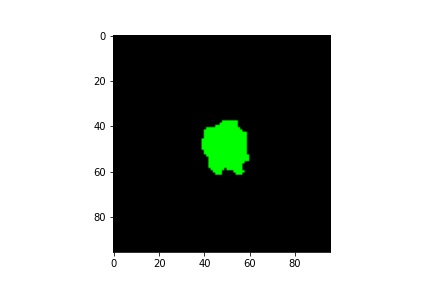

Supplement: Supplementary file 2 — Supplementary Information 2. [file 41598_2024_55178_MOESM2_ESM.zip › dcvnet/0_0_3.jpg]

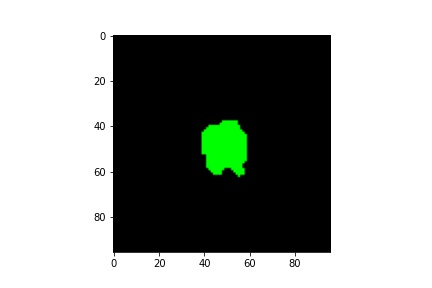

Supplement: Supplementary file 2 — Supplementary Information 2. [file 41598_2024_55178_MOESM2_ESM.zip › dcvnet/0_0_4.jpg]

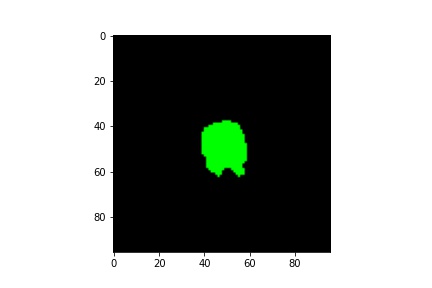

Supplement: Supplementary file 2 — Supplementary Information 2. [file 41598_2024_55178_MOESM2_ESM.zip › dcvnet/0_0_5.jpg]

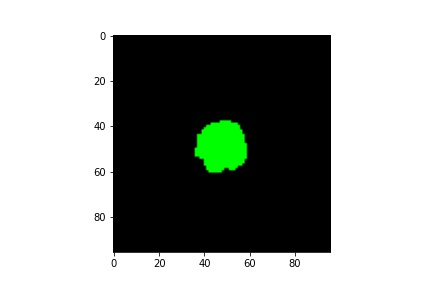

Supplement: Supplementary file 2 — Supplementary Information 2. [file 41598_2024_55178_MOESM2_ESM.zip › dcvnet/0_0_6.jpg]

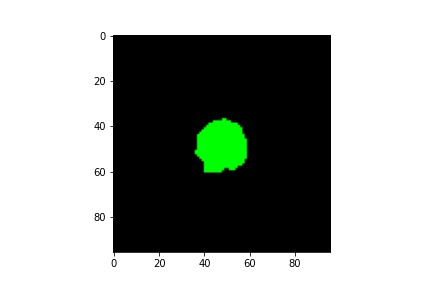

Supplement: Supplementary file 2 — Supplementary Information 2. [file 41598_2024_55178_MOESM2_ESM.zip › dcvnet/0_0_7.jpg]

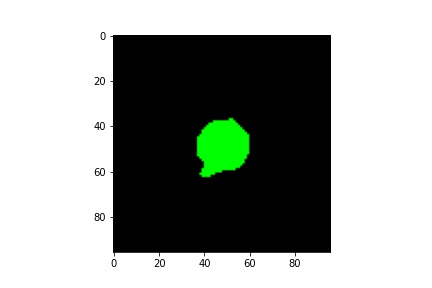

Supplement: Supplementary file 2 — Supplementary Information 2. [file 41598_2024_55178_MOESM2_ESM.zip › dcvnet/0_0_8.jpg]

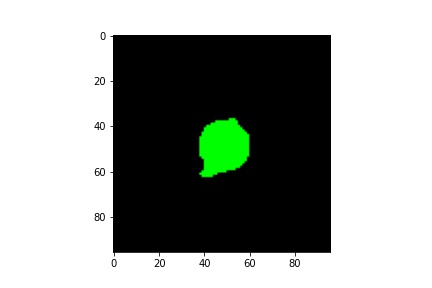

Supplement: Supplementary file 2 — Supplementary Information 2. [file 41598_2024_55178_MOESM2_ESM.zip › dcvnet/0_0_9.jpg]

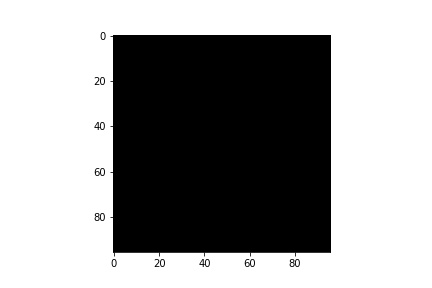

Supplement: Supplementary file 2 — Supplementary Information 2. [file 41598_2024_55178_MOESM2_ESM.zip › dcvnet/0_1_0.jpg]

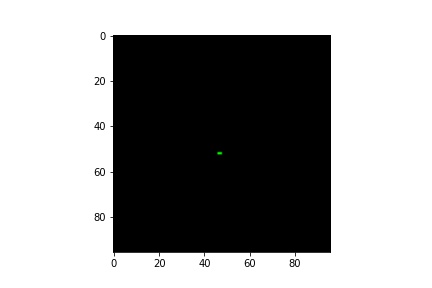

Supplement: Supplementary file 2 — Supplementary Information 2. [file 41598_2024_55178_MOESM2_ESM.zip › dcvnet/0_1_10.jpg]

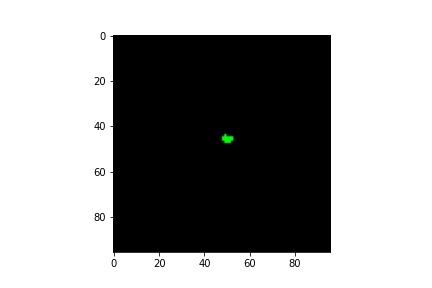

Supplement: Supplementary file 2 — Supplementary Information 2. [file 41598_2024_55178_MOESM2_ESM.zip › dcvnet/0_1_3.jpg]

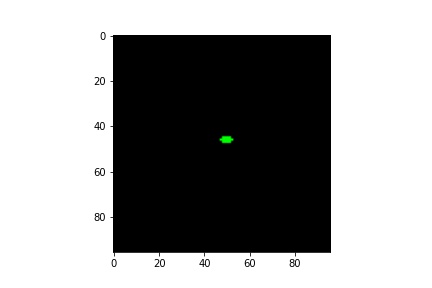

Supplement: Supplementary file 2 — Supplementary Information 2. [file 41598_2024_55178_MOESM2_ESM.zip › dcvnet/0_1_4.jpg]

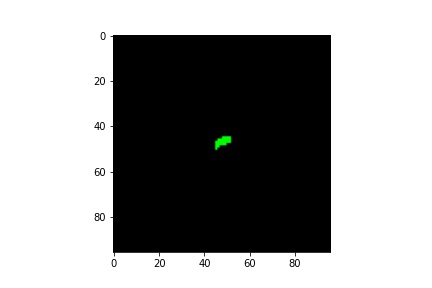

Supplement: Supplementary file 2 — Supplementary Information 2. [file 41598_2024_55178_MOESM2_ESM.zip › dcvnet/0_1_5.jpg]

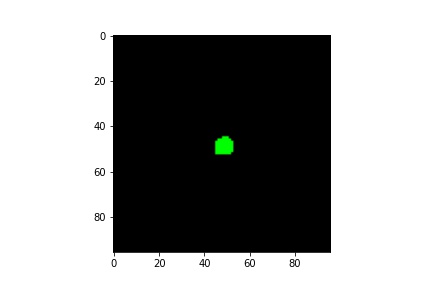

Supplement: Supplementary file 2 — Supplementary Information 2. [file 41598_2024_55178_MOESM2_ESM.zip › dcvnet/0_1_6.jpg]

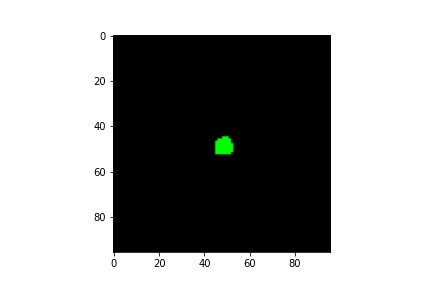

Supplement: Supplementary file 2 — Supplementary Information 2. [file 41598_2024_55178_MOESM2_ESM.zip › dcvnet/0_1_7.jpg]

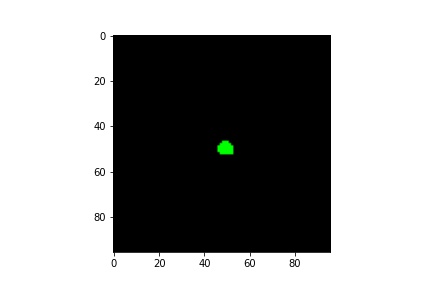

Supplement: Supplementary file 2 — Supplementary Information 2. [file 41598_2024_55178_MOESM2_ESM.zip › dcvnet/0_1_8.jpg]

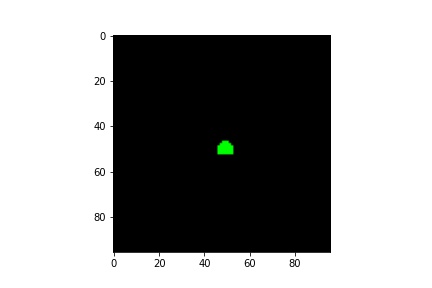

Supplement: Supplementary file 2 — Supplementary Information 2. [file 41598_2024_55178_MOESM2_ESM.zip › dcvnet/0_1_9.jpg]

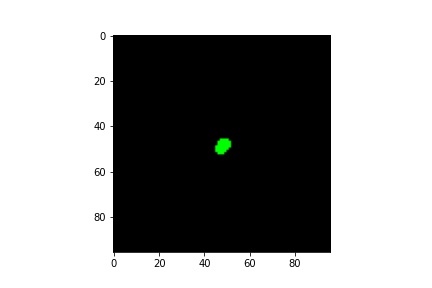

Supplement: Supplementary file 2 — Supplementary Information 2. [file 41598_2024_55178_MOESM2_ESM.zip › dcvnet/10_0_4.jpg]

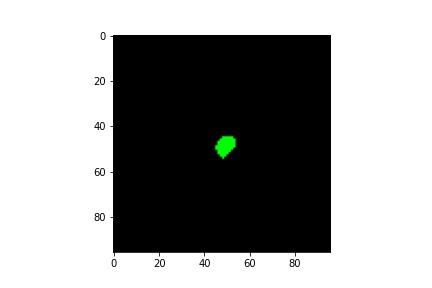

Supplement: Supplementary file 2 — Supplementary Information 2. [file 41598_2024_55178_MOESM2_ESM.zip › dcvnet/10_0_5.jpg]

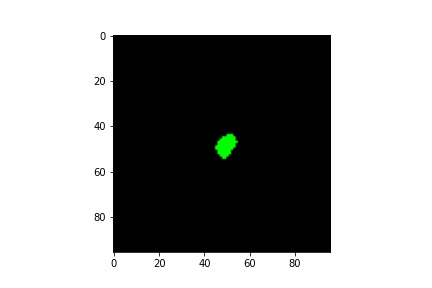

Supplement: Supplementary file 2 — Supplementary Information 2. [file 41598_2024_55178_MOESM2_ESM.zip › dcvnet/10_0_6.jpg]

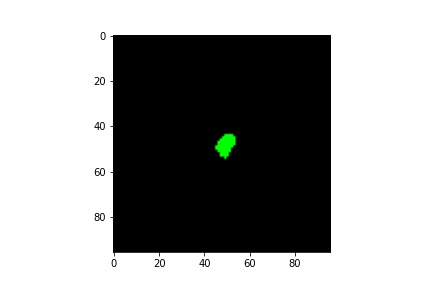

Supplement: Supplementary file 2 — Supplementary Information 2. [file 41598_2024_55178_MOESM2_ESM.zip › dcvnet/10_0_7.jpg]

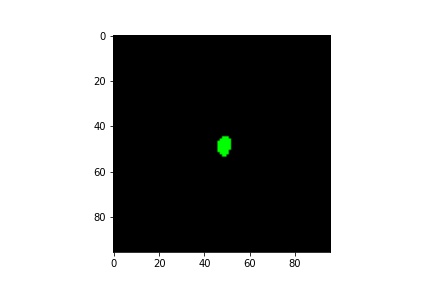

Supplement: Supplementary file 2 — Supplementary Information 2. [file 41598_2024_55178_MOESM2_ESM.zip › dcvnet/10_0_8.jpg]

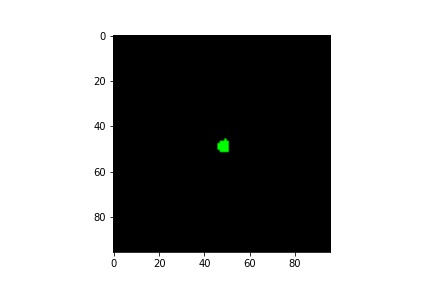

Supplement: Supplementary file 2 — Supplementary Information 2. [file 41598_2024_55178_MOESM2_ESM.zip › dcvnet/10_0_9.jpg]

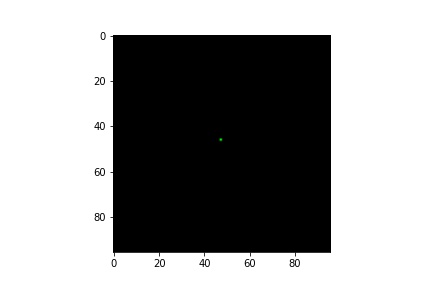

Supplement: Supplementary file 2 — Supplementary Information 2. [file 41598_2024_55178_MOESM2_ESM.zip › dcvnet/10_1_1.jpg]

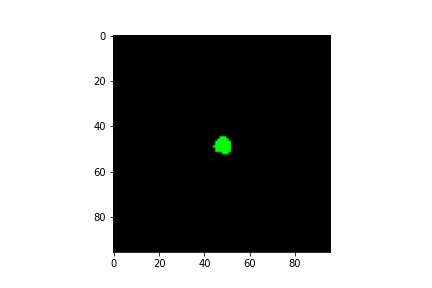

Supplement: Supplementary file 2 — Supplementary Information 2. [file 41598_2024_55178_MOESM2_ESM.zip › dcvnet/10_1_10.jpg]

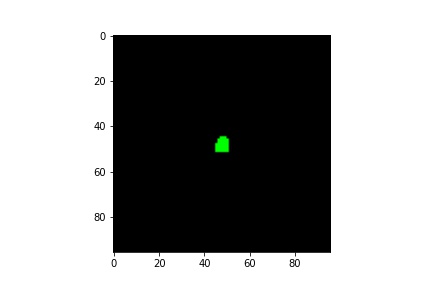

Supplement: Supplementary file 2 — Supplementary Information 2. [file 41598_2024_55178_MOESM2_ESM.zip › dcvnet/10_1_11.jpg]

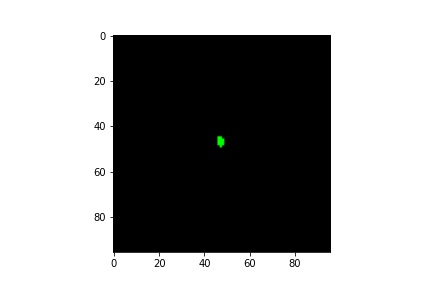

Supplement: Supplementary file 2 — Supplementary Information 2. [file 41598_2024_55178_MOESM2_ESM.zip › dcvnet/10_1_2.jpg]

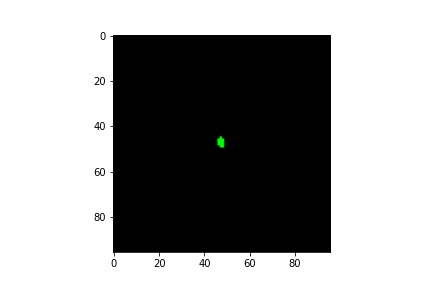

Supplement: Supplementary file 2 — Supplementary Information 2. [file 41598_2024_55178_MOESM2_ESM.zip › dcvnet/10_1_3.jpg]

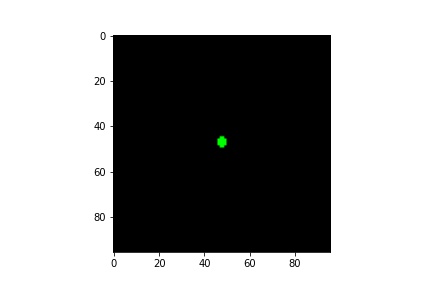

Supplement: Supplementary file 2 — Supplementary Information 2. [file 41598_2024_55178_MOESM2_ESM.zip › dcvnet/10_1_4.jpg]

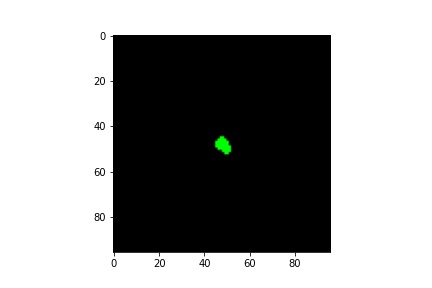

Supplement: Supplementary file 2 — Supplementary Information 2. [file 41598_2024_55178_MOESM2_ESM.zip › dcvnet/10_1_5.jpg]

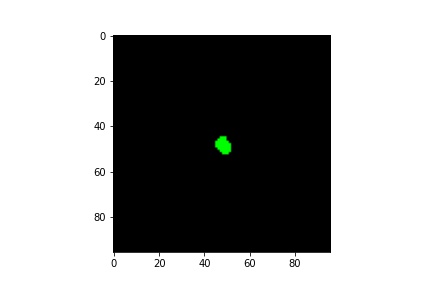

Supplement: Supplementary file 2 — Supplementary Information 2. [file 41598_2024_55178_MOESM2_ESM.zip › dcvnet/10_1_6.jpg]

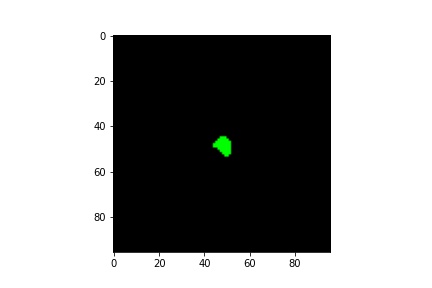

Supplement: Supplementary file 2 — Supplementary Information 2. [file 41598_2024_55178_MOESM2_ESM.zip › dcvnet/10_1_7.jpg]

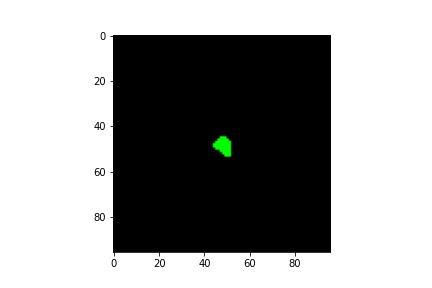

Supplement: Supplementary file 2 — Supplementary Information 2. [file 41598_2024_55178_MOESM2_ESM.zip › dcvnet/10_1_8.jpg]

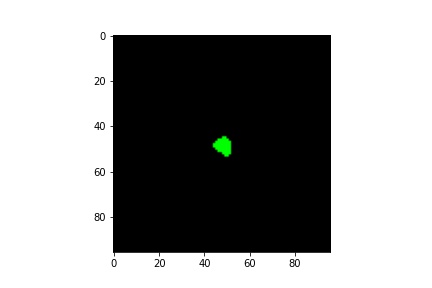

Supplement: Supplementary file 2 — Supplementary Information 2. [file 41598_2024_55178_MOESM2_ESM.zip › dcvnet/10_1_9.jpg]

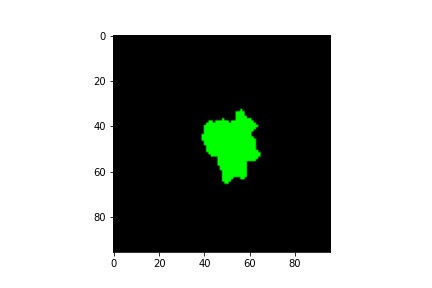

Supplement: Supplementary file 2 — Supplementary Information 2. [file 41598_2024_55178_MOESM2_ESM.zip › dcvnet/11_0_0.jpg]

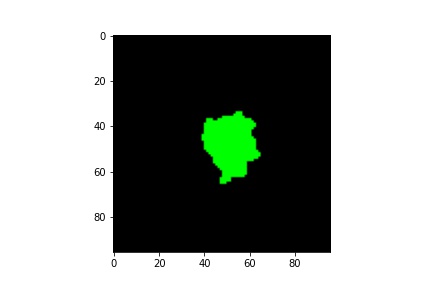

Supplement: Supplementary file 2 — Supplementary Information 2. [file 41598_2024_55178_MOESM2_ESM.zip › dcvnet/11_0_1.jpg]

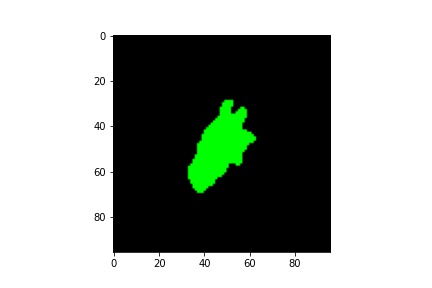

Supplement: Supplementary file 2 — Supplementary Information 2. [file 41598_2024_55178_MOESM2_ESM.zip › dcvnet/11_0_10.jpg]

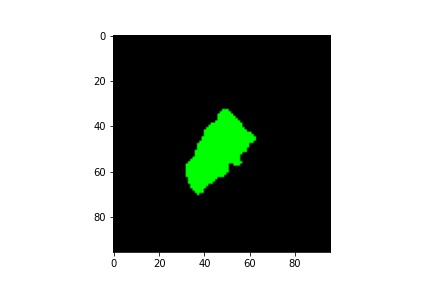

Supplement: Supplementary file 2 — Supplementary Information 2. [file 41598_2024_55178_MOESM2_ESM.zip › dcvnet/11_0_11.jpg]

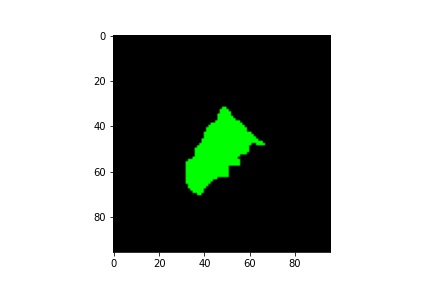

Supplement: Supplementary file 2 — Supplementary Information 2. [file 41598_2024_55178_MOESM2_ESM.zip › dcvnet/11_0_12.jpg]

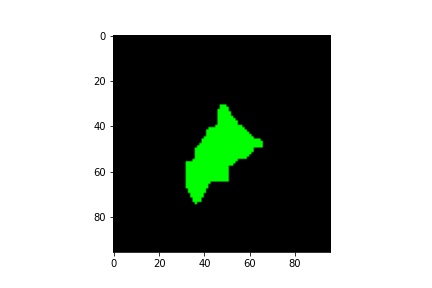

Supplement: Supplementary file 2 — Supplementary Information 2. [file 41598_2024_55178_MOESM2_ESM.zip › dcvnet/11_0_13.jpg]

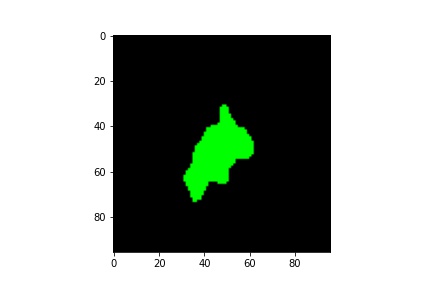

Supplement: Supplementary file 2 — Supplementary Information 2. [file 41598_2024_55178_MOESM2_ESM.zip › dcvnet/11_0_14.jpg]

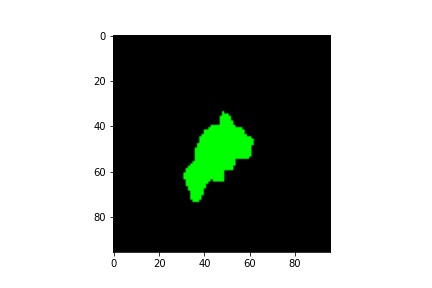

Supplement: Supplementary file 2 — Supplementary Information 2. [file 41598_2024_55178_MOESM2_ESM.zip › dcvnet/11_0_15.jpg]

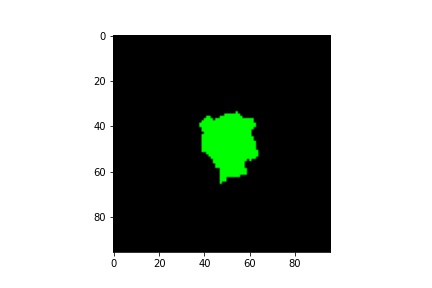

Supplement: Supplementary file 2 — Supplementary Information 2. [file 41598_2024_55178_MOESM2_ESM.zip › dcvnet/11_0_2.jpg]

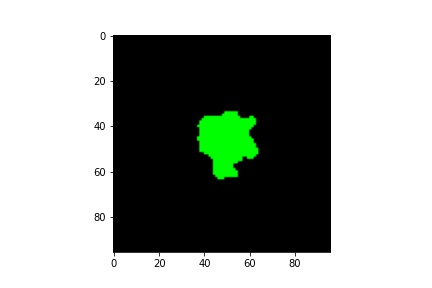

Supplement: Supplementary file 2 — Supplementary Information 2. [file 41598_2024_55178_MOESM2_ESM.zip › dcvnet/11_0_3.jpg]

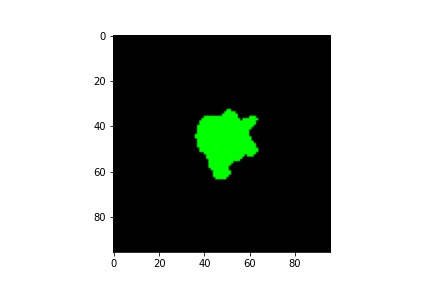

Supplement: Supplementary file 2 — Supplementary Information 2. [file 41598_2024_55178_MOESM2_ESM.zip › dcvnet/11_0_4.jpg]

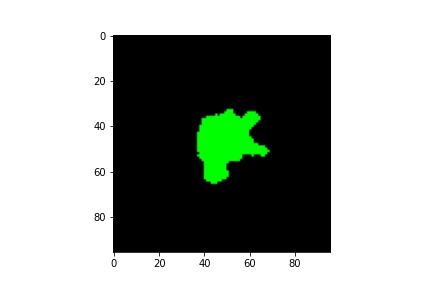

Supplement: Supplementary file 2 — Supplementary Information 2. [file 41598_2024_55178_MOESM2_ESM.zip › dcvnet/11_0_5.jpg]

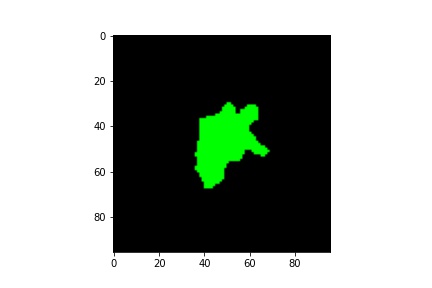

Supplement: Supplementary file 2 — Supplementary Information 2. [file 41598_2024_55178_MOESM2_ESM.zip › dcvnet/11_0_6.jpg]

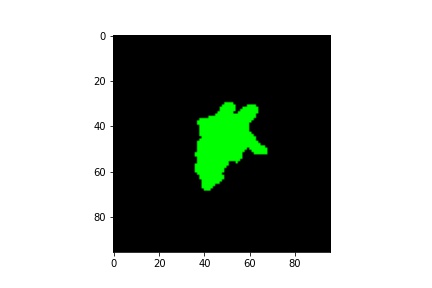

Supplement: Supplementary file 2 — Supplementary Information 2. [file 41598_2024_55178_MOESM2_ESM.zip › dcvnet/11_0_7.jpg]

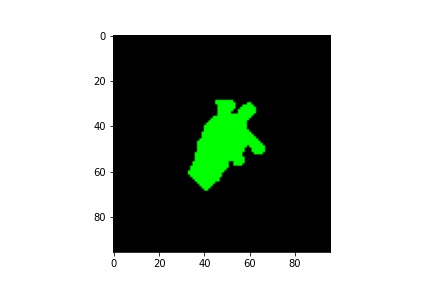

Supplement: Supplementary file 2 — Supplementary Information 2. [file 41598_2024_55178_MOESM2_ESM.zip › dcvnet/11_0_8.jpg]

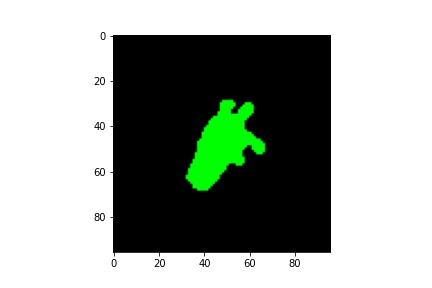

Supplement: Supplementary file 2 — Supplementary Information 2. [file 41598_2024_55178_MOESM2_ESM.zip › dcvnet/11_0_9.jpg]

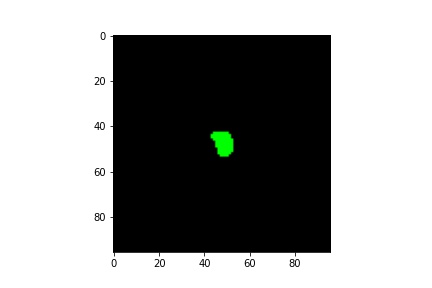

Supplement: Supplementary file 2 — Supplementary Information 2. [file 41598_2024_55178_MOESM2_ESM.zip › dcvnet/11_1_10.jpg]

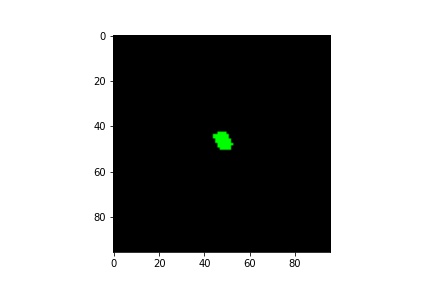

Supplement: Supplementary file 2 — Supplementary Information 2. [file 41598_2024_55178_MOESM2_ESM.zip › dcvnet/11_1_11.jpg]

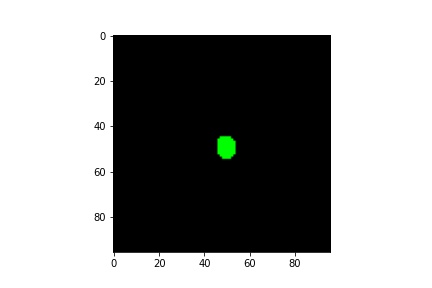

Supplement: Supplementary file 2 — Supplementary Information 2. [file 41598_2024_55178_MOESM2_ESM.zip › dcvnet/11_1_4.jpg]

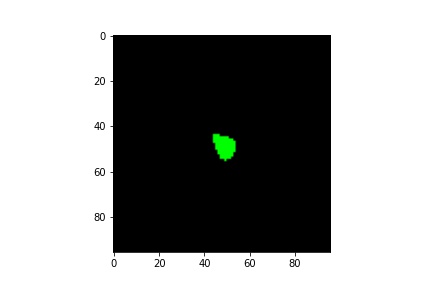

Supplement: Supplementary file 2 — Supplementary Information 2. [file 41598_2024_55178_MOESM2_ESM.zip › dcvnet/11_1_5.jpg]

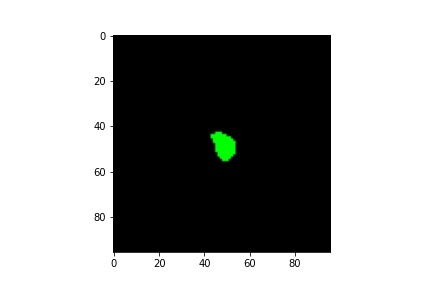

Supplement: Supplementary file 2 — Supplementary Information 2. [file 41598_2024_55178_MOESM2_ESM.zip › dcvnet/11_1_6.jpg]

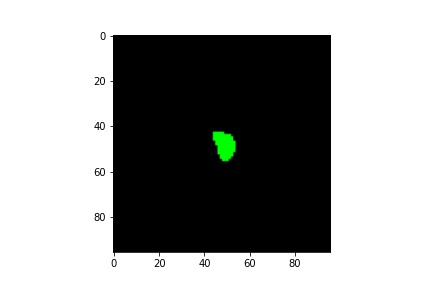

Supplement: Supplementary file 2 — Supplementary Information 2. [file 41598_2024_55178_MOESM2_ESM.zip › dcvnet/11_1_7.jpg]

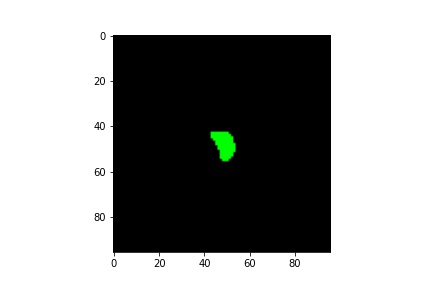

Supplement: Supplementary file 2 — Supplementary Information 2. [file 41598_2024_55178_MOESM2_ESM.zip › dcvnet/11_1_8.jpg]

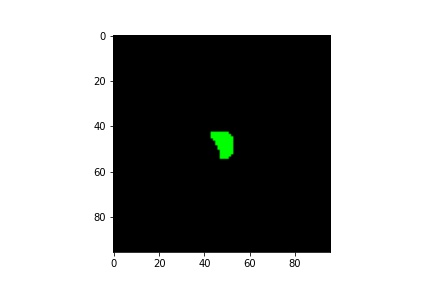

Supplement: Supplementary file 2 — Supplementary Information 2. [file 41598_2024_55178_MOESM2_ESM.zip › dcvnet/11_1_9.jpg]

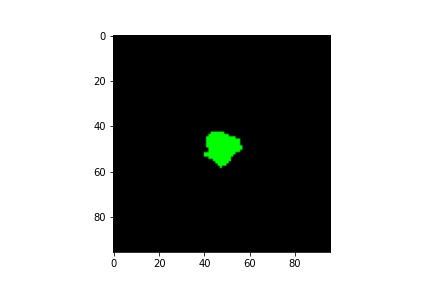

Supplement: Supplementary file 2 — Supplementary Information 2. [file 41598_2024_55178_MOESM2_ESM.zip › dcvnet/12_0_10.jpg]

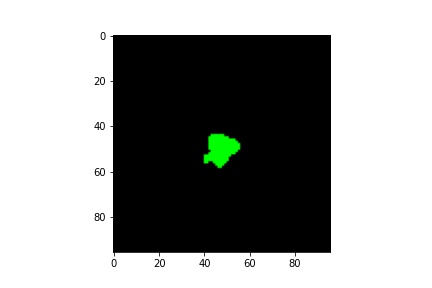

Supplement: Supplementary file 2 — Supplementary Information 2. [file 41598_2024_55178_MOESM2_ESM.zip › dcvnet/12_0_11.jpg]

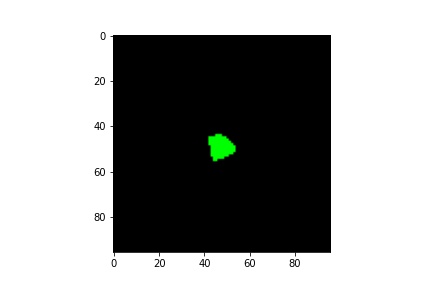

Supplement: Supplementary file 2 — Supplementary Information 2. [file 41598_2024_55178_MOESM2_ESM.zip › dcvnet/12_0_12.jpg]

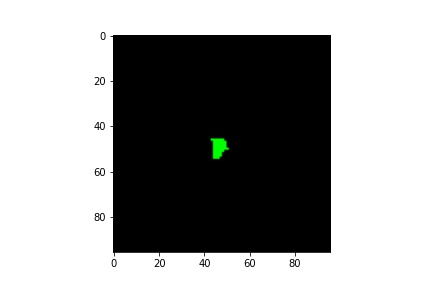

Supplement: Supplementary file 2 — Supplementary Information 2. [file 41598_2024_55178_MOESM2_ESM.zip › dcvnet/12_0_13.jpg]

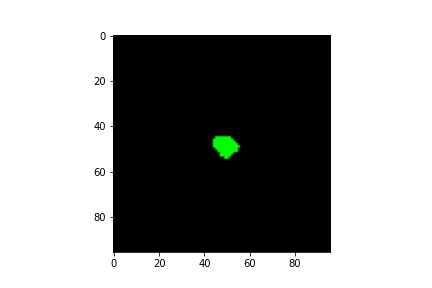

Supplement: Supplementary file 2 — Supplementary Information 2. [file 41598_2024_55178_MOESM2_ESM.zip › dcvnet/12_0_3.jpg]

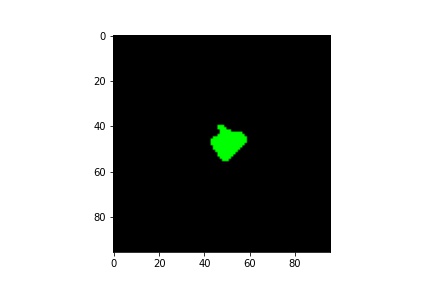

Supplement: Supplementary file 2 — Supplementary Information 2. [file 41598_2024_55178_MOESM2_ESM.zip › dcvnet/12_0_4.jpg]

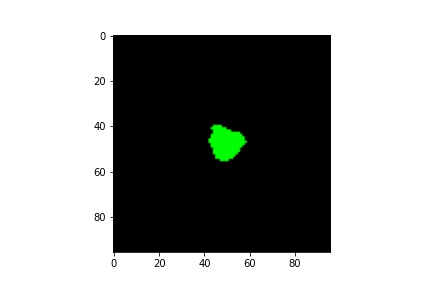

Supplement: Supplementary file 2 — Supplementary Information 2. [file 41598_2024_55178_MOESM2_ESM.zip › dcvnet/12_0_5.jpg]

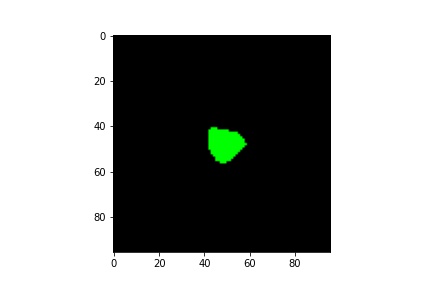

Supplement: Supplementary file 2 — Supplementary Information 2. [file 41598_2024_55178_MOESM2_ESM.zip › dcvnet/12_0_6.jpg]

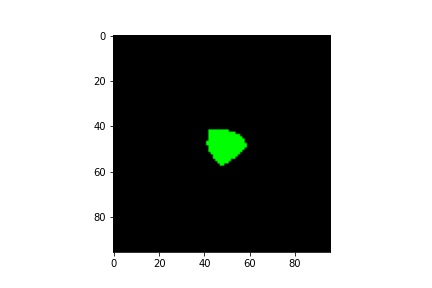

Supplement: Supplementary file 2 — Supplementary Information 2. [file 41598_2024_55178_MOESM2_ESM.zip › dcvnet/12_0_7.jpg]

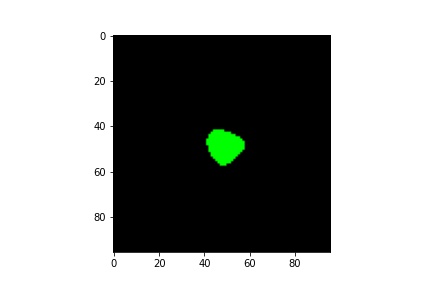

Supplement: Supplementary file 2 — Supplementary Information 2. [file 41598_2024_55178_MOESM2_ESM.zip › dcvnet/12_0_8.jpg]

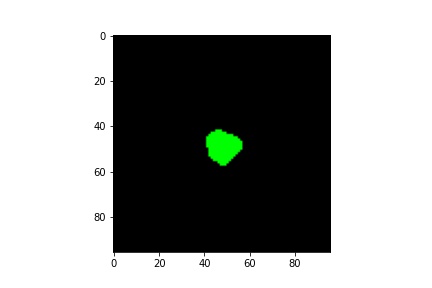

Supplement: Supplementary file 2 — Supplementary Information 2. [file 41598_2024_55178_MOESM2_ESM.zip › dcvnet/12_0_9.jpg]

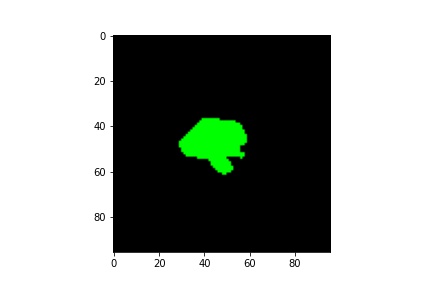

Supplement: Supplementary file 2 — Supplementary Information 2. [file 41598_2024_55178_MOESM2_ESM.zip › dcvnet/12_1_0.jpg]

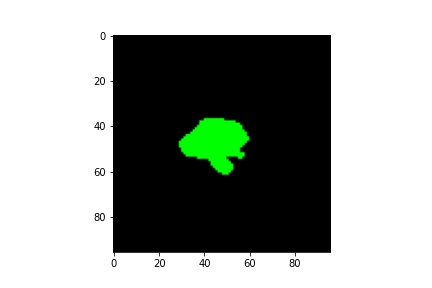

Supplement: Supplementary file 2 — Supplementary Information 2. [file 41598_2024_55178_MOESM2_ESM.zip › dcvnet/12_1_1.jpg]

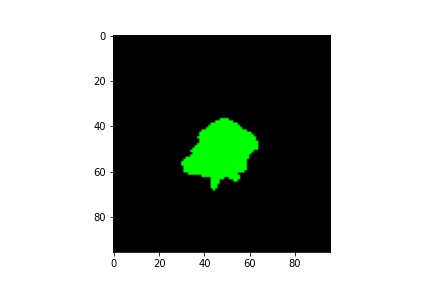

Supplement: Supplementary file 2 — Supplementary Information 2. [file 41598_2024_55178_MOESM2_ESM.zip › dcvnet/12_1_10.jpg]

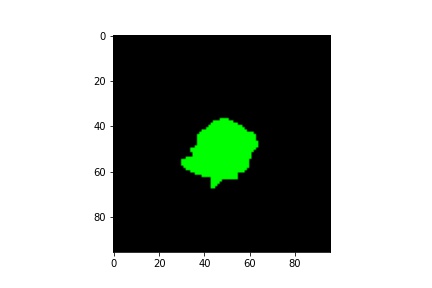

Supplement: Supplementary file 2 — Supplementary Information 2. [file 41598_2024_55178_MOESM2_ESM.zip › dcvnet/12_1_11.jpg]

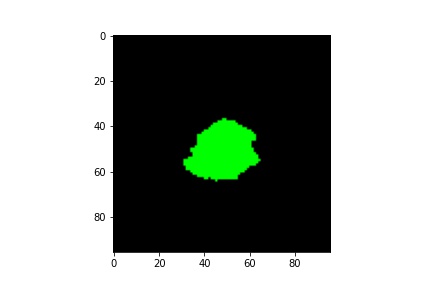

Supplement: Supplementary file 2 — Supplementary Information 2. [file 41598_2024_55178_MOESM2_ESM.zip › dcvnet/12_1_12.jpg]

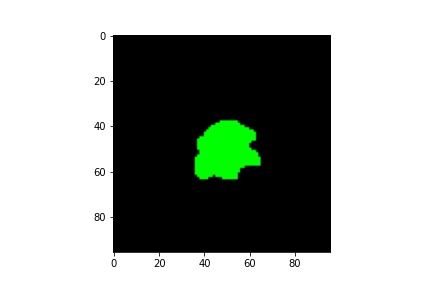

Supplement: Supplementary file 2 — Supplementary Information 2. [file 41598_2024_55178_MOESM2_ESM.zip › dcvnet/12_1_13.jpg]

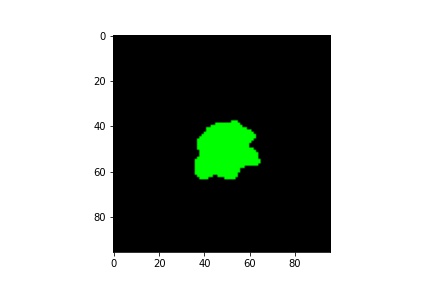

Supplement: Supplementary file 2 — Supplementary Information 2. [file 41598_2024_55178_MOESM2_ESM.zip › dcvnet/12_1_14.jpg]

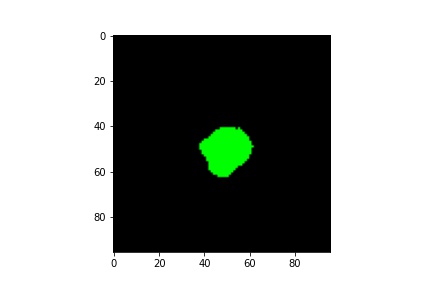

Supplement: Supplementary file 2 — Supplementary Information 2. [file 41598_2024_55178_MOESM2_ESM.zip › dcvnet/12_1_15.jpg]

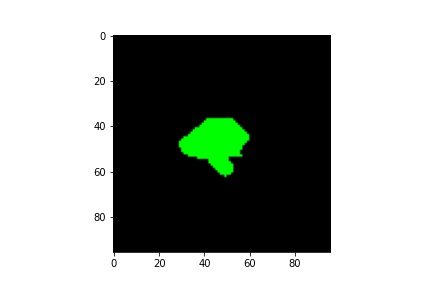

Supplement: Supplementary file 2 — Supplementary Information 2. [file 41598_2024_55178_MOESM2_ESM.zip › dcvnet/12_1_2.jpg]

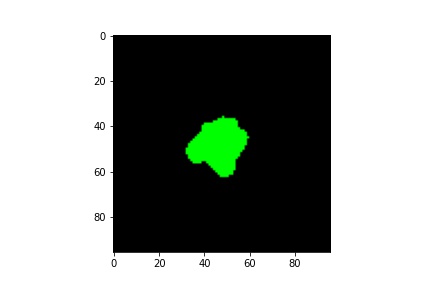

Supplement: Supplementary file 2 — Supplementary Information 2. [file 41598_2024_55178_MOESM2_ESM.zip › dcvnet/12_1_3.jpg]

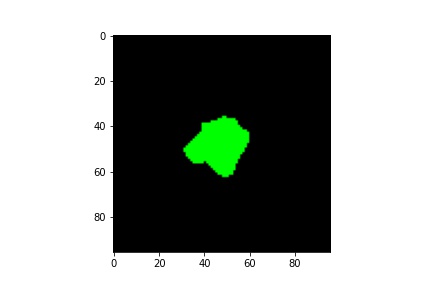

Supplement: Supplementary file 2 — Supplementary Information 2. [file 41598_2024_55178_MOESM2_ESM.zip › dcvnet/12_1_4.jpg]

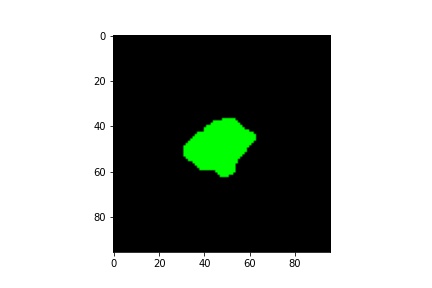

Supplement: Supplementary file 2 — Supplementary Information 2. [file 41598_2024_55178_MOESM2_ESM.zip › dcvnet/12_1_5.jpg]

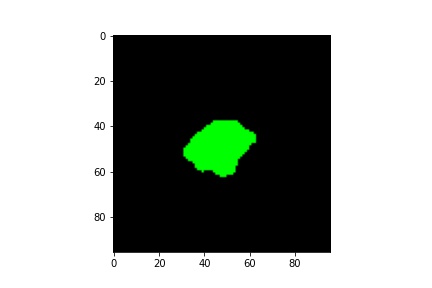

Supplement: Supplementary file 2 — Supplementary Information 2. [file 41598_2024_55178_MOESM2_ESM.zip › dcvnet/12_1_6.jpg]

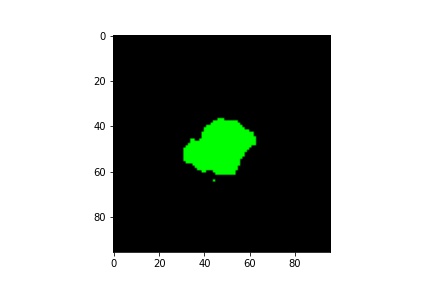

Supplement: Supplementary file 2 — Supplementary Information 2. [file 41598_2024_55178_MOESM2_ESM.zip › dcvnet/12_1_7.jpg]

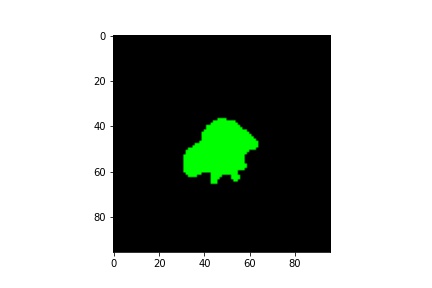

Supplement: Supplementary file 2 — Supplementary Information 2. [file 41598_2024_55178_MOESM2_ESM.zip › dcvnet/12_1_8.jpg]

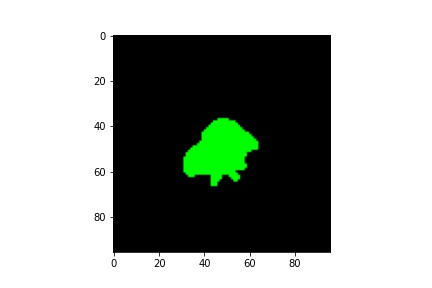

Supplement: Supplementary file 2 — Supplementary Information 2. [file 41598_2024_55178_MOESM2_ESM.zip › dcvnet/12_1_9.jpg]

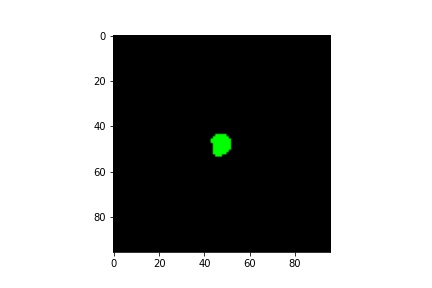

Supplement: Supplementary file 2 — Supplementary Information 2. [file 41598_2024_55178_MOESM2_ESM.zip › dcvnet/13_0_10.jpg]

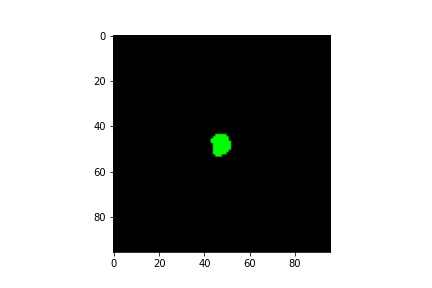

Supplement: Supplementary file 2 — Supplementary Information 2. [file 41598_2024_55178_MOESM2_ESM.zip › dcvnet/13_0_11.jpg]

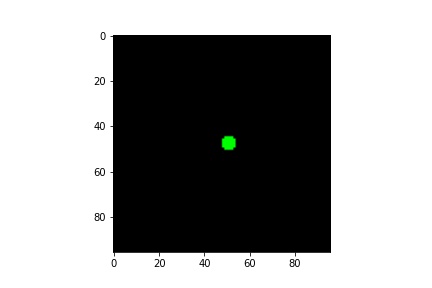

Supplement: Supplementary file 2 — Supplementary Information 2. [file 41598_2024_55178_MOESM2_ESM.zip › dcvnet/13_0_2.jpg]

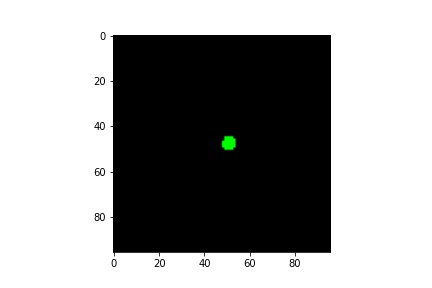

Supplement: Supplementary file 2 — Supplementary Information 2. [file 41598_2024_55178_MOESM2_ESM.zip › dcvnet/13_0_3.jpg]

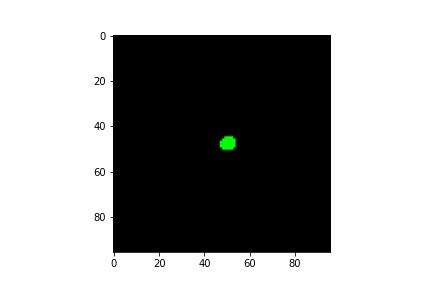

Supplement: Supplementary file 2 — Supplementary Information 2. [file 41598_2024_55178_MOESM2_ESM.zip › dcvnet/13_0_4.jpg]

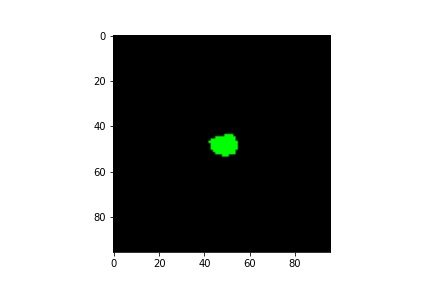

Supplement: Supplementary file 2 — Supplementary Information 2. [file 41598_2024_55178_MOESM2_ESM.zip › dcvnet/13_0_5.jpg]

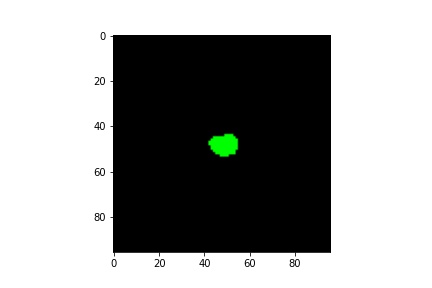

Supplement: Supplementary file 2 — Supplementary Information 2. [file 41598_2024_55178_MOESM2_ESM.zip › dcvnet/13_0_6.jpg]
